# Metastable exohedrally decorated Borospherene B_40_

**DOI:** 10.1038/s41598-017-06877-7

**Published:** 2017-08-08

**Authors:** Santanu Saha, Luigi Genovese, Stefan Goedecker

**Affiliations:** 1 Department of Physics, Universität Basel, Klingelbergstrasse 82, 4056 Basel, Switzerland; 2 Université Grenoble Alpes, Institut Nanosciences et Cryogénie-Modélisation et Exploration des Matériaux, L_Sim, F-38000 Grenoble, France

## Abstract

The experimental discovery of borospherene, the only non-carbon fullerene observed in nature, has generated a lot of interest in the scientific community and led to the theoretical prediction of various endohedrally and exohedrally decorated borospherene. We apply Minima Hopping Method (MHM), a global geometry optimization algorithm at the density functional level to check the stability of recently proposed exohedrally decorated borospherenes M_6_@B_40_ for (M = Li, Na, K, Rb, Be, Mg, Ca, Sr, Sc and Ti). By performing short MHM runs, we find that the proposed fullerene structures are not global minima. Our new lowest energy structures are significantly deformed and of much lower symmetry. These low energy structures spontaneously aggregate by forming chemical bonds when they are brought together. Therefore, it would be challenging to synthesize bulk materials made out of the theoretically postulated exohedrally decorated borospherenes such as B_40_M_6_ which might have technologically useful properties.

## Introduction

Considerable theoretical efforts are under way in nanosciences to find fullerene structures made out of non-carbon materials and numerous theoretical non-carbon structures can be found in the literature^1–5^. However, up to date nearly all experimentally discovered structures^6–15^ are either pure carbon systems or are based on carbon fullerene skeletons, which are decorated by other elements or where some carbon atoms are replaced by other elements. Several theoretically proposed structures were later shown to be metastable and to be much higher in energy than the ground state. As a consequence it is very unlikely that such structures could ever be synthesized. This was for instance the case for endohedrally doped Si_20_ fullerene^16^ and the B_80_ fullerene^17–19^. A boron-carbon heterofullerene with boron patches was found to be the ground state instead of a configuration where the boron atoms are homogeneously distributed^4^.

Boron (B_*n*_) cages of different sizes have been proposed theoretically (n = 28 and 38)^20–22^. After two decades of search a cage structure for (${{\rm{B}}}_{40}^{-1}$) was finally observed experimentally by Zhai *et al*.^23^ together with a quasi planar structure. Calculations gave a lower energy to the quasi planar structure. However, according to the same kind of theoretical calculations, the ground state of neutral B_40_ has a cage like structure. It has a fullerene structure with *D*_2*d*_ symmetry consisting of two planar hexagonal and four non-planar heptagonal rings. This ground state structure is 0.5 eV lower in energy than the second minimum^23^. The quasi planar structure is fifth lowest in energy with an energy difference of 1 eV. The discovery of borospherene has generated a lot of interest in the scientific community. An important difference to C_60_ has however to be noted. Carbon fullerenes attract each other only by weak van der Waals forces, but do not form covalent bonds among each other. As already noted by Zhai *et al*.^23^, B_40_ is expected to be highly reactive^17, 24^, and should therefore form covalent bonds with adjacent borospherenes, destroying its original fullerene shape. The results discussed in ref. 25 and presented in this article confirm this expectation. Soon after the discovery of B_40_, ${{\rm{B}}}_{39}^{-}$ an axially chiral borospherene was discovered by Chen *et al*.^26^.

Since the discovery of borospherene, a large number of decorated borospherene structures have been proposed theoretically primarily for applications in hydrogen storage. The decoration of B_40_ can be classified by the type of adsorbate atoms (adatoms), which can be an alkaline metal, earth alkaline metal or transition metal. Bai *et al*.^27^ performed minima hopping based structure predictions for endohedral M@B_40_ (M = Ca, Sr) and exohedral M@B_40_ (M = Be, Mg). Fa *et al*.^28^ found endohedral M@B_40_ (M = Na, Ba) to be stable. Jin. *et al*.^29^ investigated endohedral M@B_40_ (M = Sc, Y, La) and observed that they have strong binding energies and may thus exist in nature. Hydrogen adsorption studies on Li decorated B_40_ by Bai *et al*.^30^ predict that H_2_ storage can be increased from 7.1 wt% in Li_6_B_40_ to 13.8 wt% in Li_14_B_40_. The theoretical studies of Liu *et al*.^31^ showed that exohedrally decorated B_40_ with six alkaline metal atoms (AM = Li, Na, K) is stable and could achieve a hydrogen storage capacity of 8 wt%. Tang *et al*.^32^ predicted Sc decorated B_40_ to be stable through short molecular dynamics simulation. Based on a study of single metal atom adsorption energies, Dong *et al*.^33^ proposed a B_40_ fullerene decorated with six Ti atoms as a promising candidate for hydrogen storage. In all the above mentioned theoretical B_40_ M_6_ structures, the six adatoms are centered in the two hexagons and four heptagons of the bare B_40_ fullerene.

However, in none of these later studies systematic structure predictions were performed. By performing structure predictions, we will show in this contribution, that the B_40_ fullerene decorated with six alkaline metal, earth alkaline metal or transition metal atoms is only a metastable structure and that there are other disordered structures which are considerably lower in energy. These low energy structures are in addition highly reactive and form bonds when brought into contact with each other. Hence it would be extremely difficult to synthesize bulk materials made of metal decorated B_40_ fullerene building blocks.

## Results and Discussions

In this work we study the potential energy surface of borospherene M_6_@B_40_ decorated with six metal atoms. We consider alkaline metals (Li, Na, K and Rb), earth alkaline metals (Be, Mg, Ca and Sr) and transition metals (TM = Sc and Ti) in our study.

**Figure 1:**
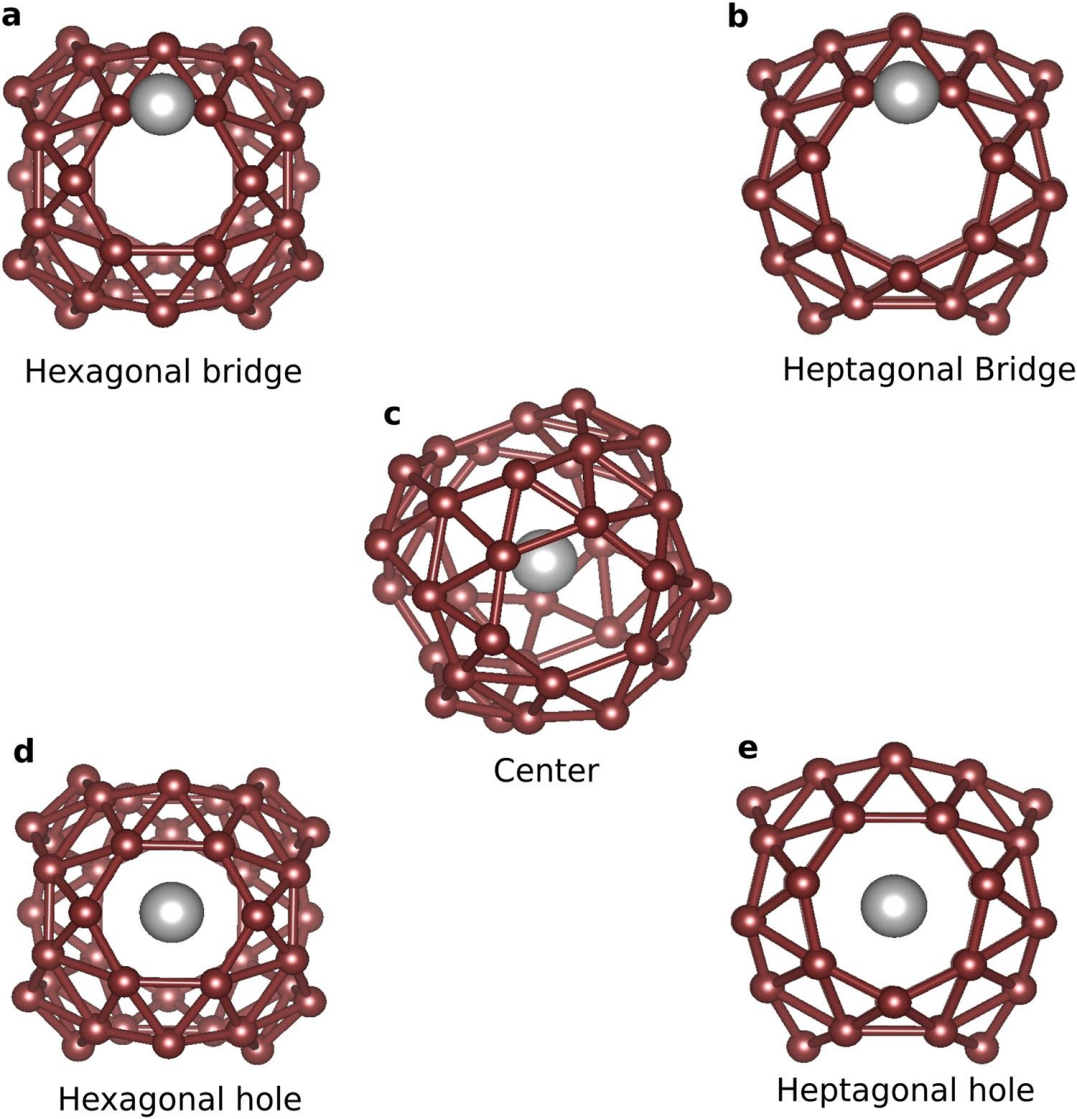
The adsorbate atom, represented by grey ball, can be placed on five different positions in the borospherene (B_40_) represented by brown balls. The five different sites are (**a**) on the B-B bond of the hexagonal ring, (**b**) on the B-B bond of the heptagonal ring, (**c**) center of the cage, (**d**) hexagonal hole and (**e**) heptagonal hole.

Before discussing the six-atom decorations of the B_40_ cage, let us briefly address the single adatom case. A single adatom can sit in the center of the cage, centers of the hexagons and heptagons or on the B-B bridge of the hexagonal/heptagonal rings as shown in Fig. 1. The binding energy (B.E.) of a single adatom on B_40_ for different metal atoms and for different positions are obtained using the following formula:

1 $${E}_{bind}=-[{E}_{M@B40}-{E}_{M}-{E}_{B40}]$$

where E_*M*_ represents the energy of an isolated metal adatom, E_*B*40_ the energy of an isolated B_40_ cage and E_*M@B*40_ the energy of the decorated cage. The B.E. trends for different adatoms at different positions are shown in Table 1. During a geometry relaxation Be moves outside the cage. Li, Mg, Sc and Ti take on an off center position inside the cage and come close to either hexagon or heptagon holes whereas Na, K, Rb, Ca and Sr are stable at the center of the cage (see Supplementary Information). The alkaline metals and earth metals are unstable at the B-B bridge of the hexagon/heptagon. Upon relaxation the adatom positioned on the B-B bridge moves to the hexagon/heptagon holes. A Ti atom is stable on both the hexagonal and heptagonal B-B bridge whereas Sc is stable only on heptagonal B-B bridge. Among the earth alkaline metals, the hexagonal hole is energetically more favorable for Be and Mg whereas for Ca and Sr, the most stable site is the center of the borospherene. All the alkaline metals are most stable at the heptagonal hole except Na which is most stable at the center. This anomalous behavior of Na can be associated to two main factors: complete transfer of charge from the decorated atom to the cage and contraction of the cage due to intake of charge. The contraction leads to reduction of empty space inside the cage. The resultant empty space is too large for Li and too small for K and Rb. But, it is just perfect for Na. Hence, Na is most stable at the center of the cage. The transition metals (Sc and Ti) are most stable at the off-center position inside the borospherene cage.

**Table 1:** The binding energy (in eV) of the single adatom to different sites of the B_40_ for the PBE exchange correlation fuctional.

	CENTER (eV)	HEX (eV)	HEPT (eV)	HEX B-B (eV)	HEPT B-B (eV)
Be@B_40_	—	3.168	3.141	—	—
Mg@B_40_	0.069	1.093	1.050	—	—
Ca@B_40_	3.776	2.520	2.655	—	—
Sr@B_40_	13.167	11.666	11.746	—	—
Li@B_40_	1.796	2.112	2.254	—	—
Na@B_40_	1.683	1.546	1.582	—	—
K@B_40_	1.675	1.714	1.764	—	—
Rb@B_40_	1.017	1.670	1.714	—	—
Sc@B_40_	5.171	3.906	4.543	—	2.091
Ti@B_40_	9.466	9.089	9.257	6.815	6.775

These calculations have been carried out using BigDFT for free boundary conditions. The headings CENTER, HEX and HEPT represents the center, hexagonal hole and heptagonal hole of the borospherene respectively. HEX B-B and HEPT B-B represent the B-B bond belonging to the hexagon and heptagon ring respectively.

From the B.E. trends, it is clear that the favorable sites for binding are the center of the cage and the hexagonal/heptagonal holes of the cage. Most of the adatoms are unstable at the B-B bridges. In this work we are interested in exohedral decoration of B_40_ cages. The maximum number of suitable sites is six (2 hexagonal hole and 4 heptagonal holes). As in most cases, it is difficult to predict other possible structures by chemical intuition. Hence, in order to check the stability of M_6_@B_40_ cages and find new possible structures we use a systematic and unbiased structure prediction method, namely the Minima Hopping Method (MHM)^34–38^ as implemented in the BigDFT^39^ package. The PBE functional^40^ is used in all these runs.

**Figure 2:**
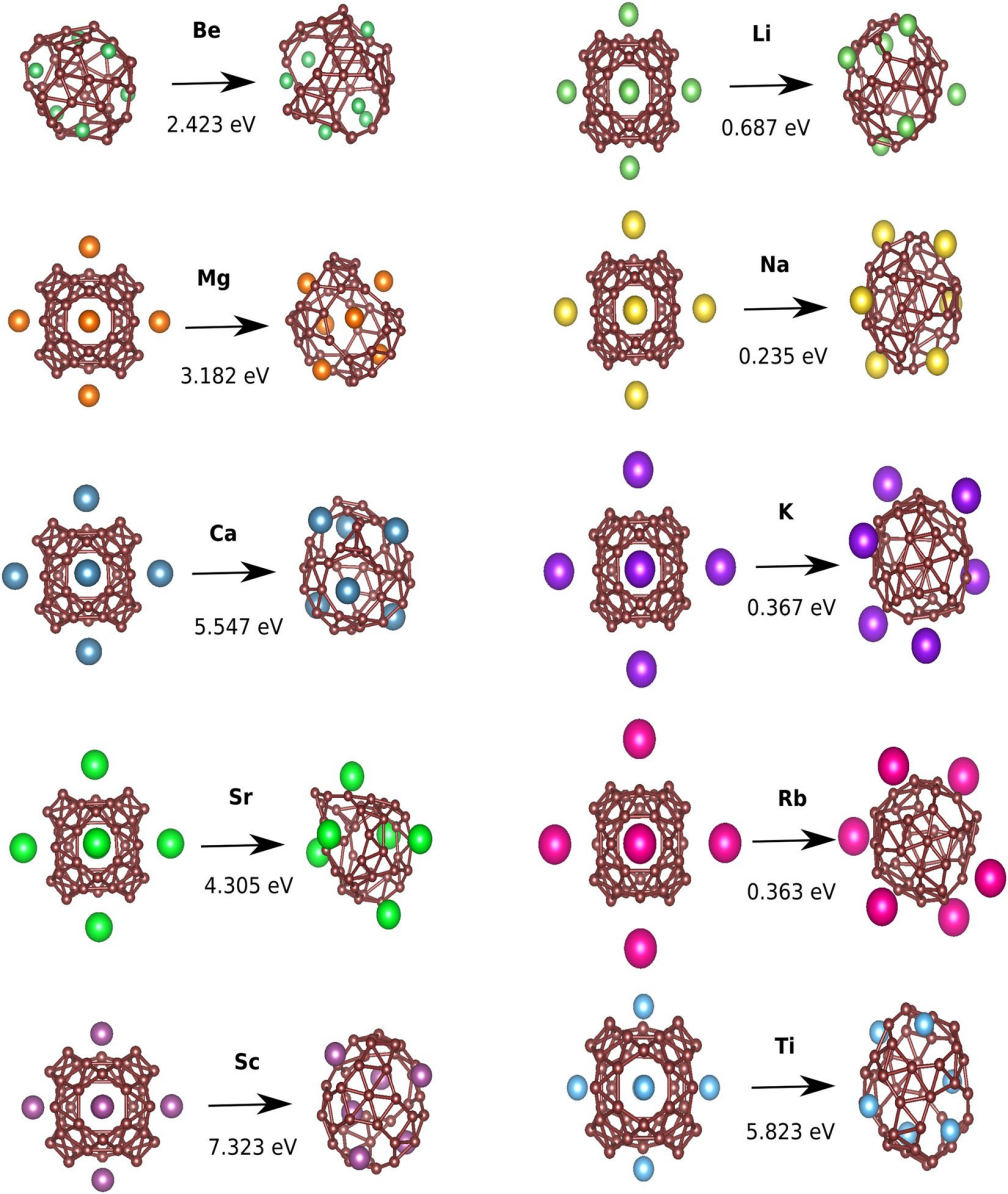
The first and third columns show the perfectly decorated cage structures of borospherene with different adsorbates: alkaline, earth alkaline and transition metals. The second and fourth columns represent the lowest energy structure found in minima hopping runs. Brown balls represent boron atoms and the other colours various metal atoms. The energy difference (in eV) between the lowest energy structure and the initial decorated borospherene is also shown for PBE exchange correlation functional.

As an input guess for the MHM runs, we placed the adatoms in the hexagonal and heptagonal centers of the perfect borospherene as these prototype structures were found to be stable in recent publications^30, 31, 33^. All the MHM runs gave very soon deformed cages that were lower in energy than the initial fullerene structure. The deformed structures often lost their characteristic hexagon-heptagon patterns and are shown in Fig. 2. These deformed structures have rings containing between 6 and 10 boron atoms. The initial guess structure of adatoms on hexagonal and heptagonal rings of borospherene were found to be local minima except for Be. For earth alkaline metals, i.e. Mg, Ca, Sr the lowest energy structures had randomly arranged rings with 7–10 atoms. In case of alkaline metals, the lowest energy structures of Li, Na, K and Rb decorating B_40_ had rings with 6–8 borons. The Sc and Ti decorated lowest energy structures had rings with 5–9 borons. The charge transfer of the decorating elements to the cage and the lowest energy structure obtained through Bader charge analysis are listed in Table 2. The trends in charge transfer are identical in both the cage structure and the lowest energy structure for each corresponding adatom. This indicates that in both cases, the adatoms have a similar kind of bonding with the B atoms.

**Table 2:** The charge transfer of the decorating atoms in the borospherene cage structure and the lowest energy structure.

	CAGE	LOW
Be@B_40_	2.00	2.00
Mg@B_40_	1.68 to 2.00	2.00
Ca@B_40_	1.20 to 1.30	1.36 to 1.40
Sr@B_40_	1.19 to 1.30	1.36 to 1.42
Li@B_40_	1.00	1.00
Na@B_40_	1.00	1.00
K@B_40_	0.75 to 0.78	0.77 to 0.83
Rb@B_40_	0.73 to 0.77	0.76 to 0.83
Sc@B_40_	1.26 to 1.44	1.56 to 1.64
Ti@B_40_	1.21 to 1.36	1.44 to 1.52

**Figure 3:**
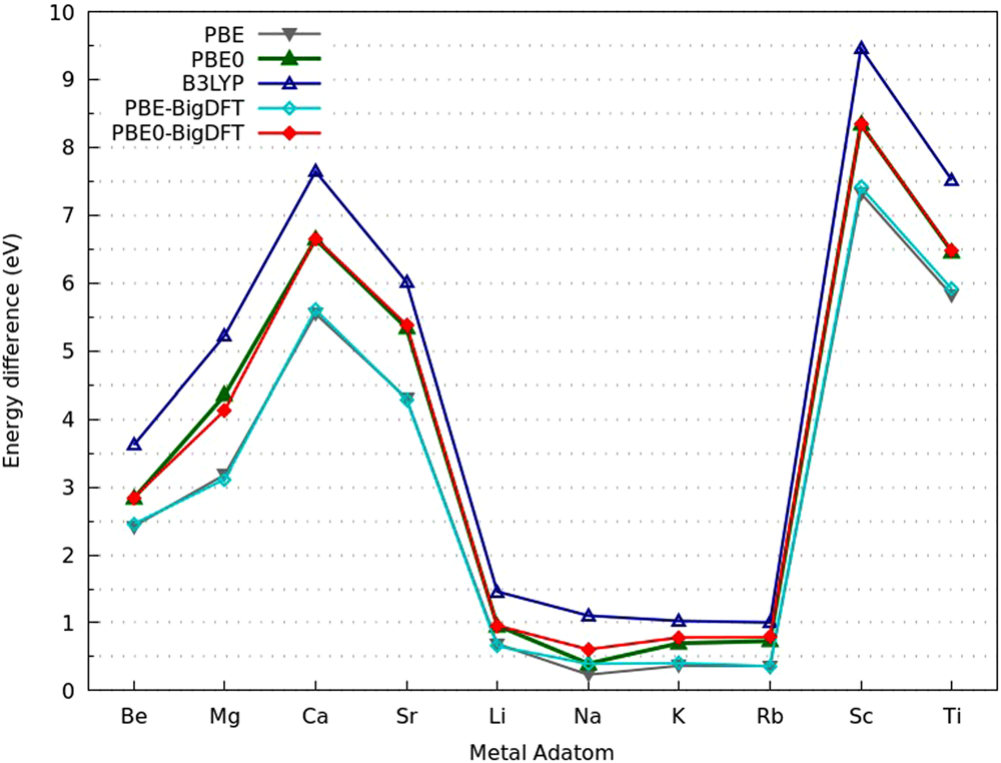
The energy difference (in eV) of the perfect cage with respect to the lowest energy structure of M_6_@B_40_ for different adatoms calculated with different exchange correlation functionals and two different codes, namely FHI-aims and BigDFT. Unless specified the calculations were done with the FHIaims code.

In ref. 41 it was shown that the PBE functional gives a reasonable description of boron clusters, which however cannot always predict the correct energetic ordering between different structures. For this reason the stability of the deformed cages was further assessed by recalculating the energy differences between the lowest energy structure found and the perfectly decorated cage structure with the PBE0^42^ and B3LYP^43–46^ functionals using the all electron FHIaims^47^ code. We also compared on the PBE level the pseudopotential results obtained from BigDFT with the all electron results obtained with FHIaims and found that they are in close agreement. The energy difference between the lowest energy structure and perfect fullerene structure agree within 200 meV. The data of Fig. 3 show that also the two other functionals predict our structures found on the PBE level to be lower in energy than the perfect cage. This indicates that even in gas-phase, it is unlikely to get an intact fullerene structure.

It is also interesting to notice that clustering of the metal atoms was never observed during our short MHM runs. This may be explained by the relatively strong binding of the metal atoms to the boron skeleton. This is in contrast to the case of C_60_ where lower binding energies lead to clustering.

**Figure 4:**
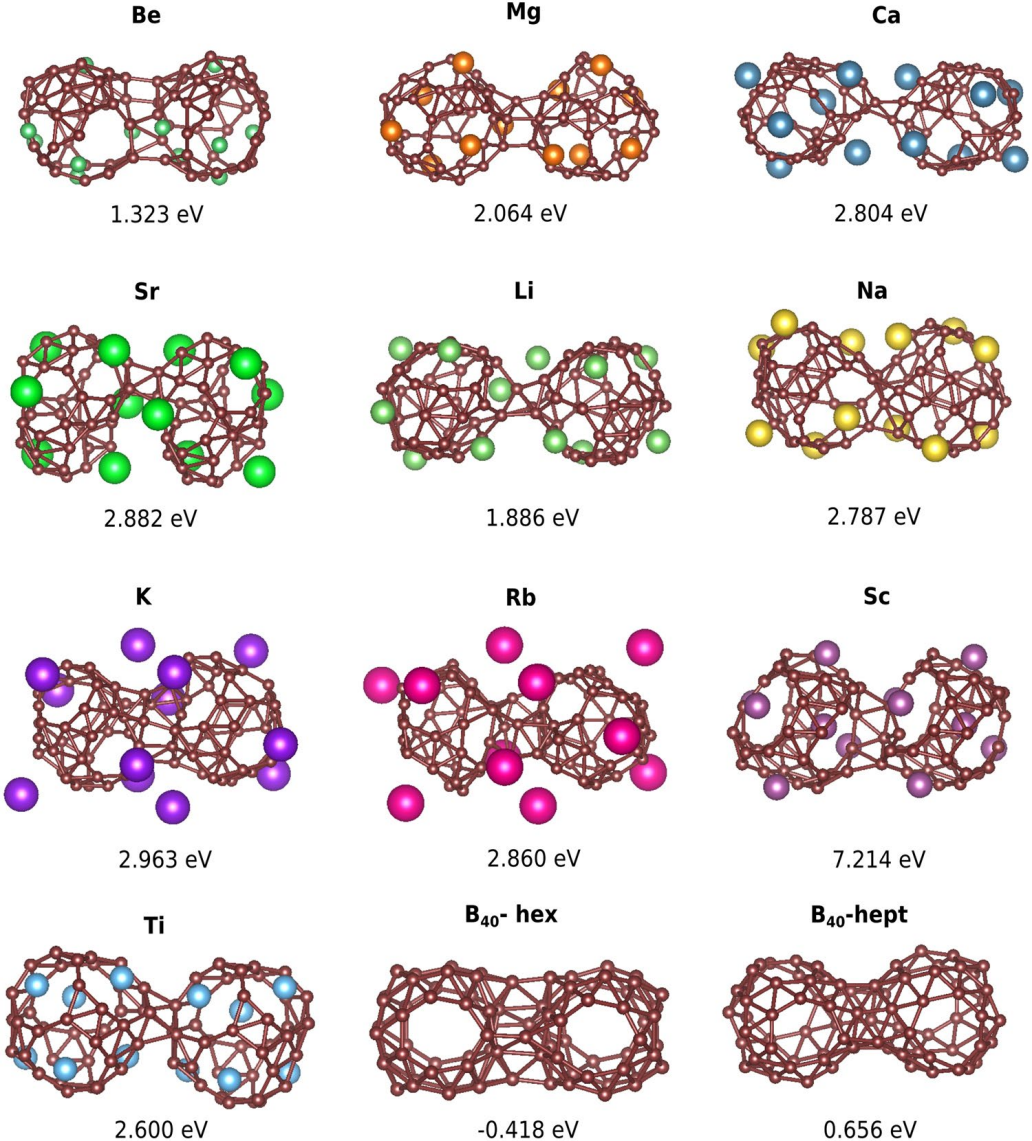
The relaxed structures of dimers made from the lowest energy structures of different M_6_B_40_’s and B_40_’s together with their dimerization energy (in eV). All the calculation have been done using BigDFT and the PBE exchange correlation functional. B_40_-hex represents dimer formed along the hexagonal rings and B_40_-hept represents dimer formed along heptagonal rings.

To study the reactivity of the deformed metal decorated B_40_ cages we brought two units in close contact and performed a geometry optimization to obtain the dimerization energy which is defined as

2 $${E}_{DE}=-[{E}_{dimer}-2{E}_{mono}]$$

where E_*mono*_ and E_*dimer*_ are the total energies of the monomer and dimer respectively. Here the monomers are the lowest energy structures. The dimerization energies (D.E.) for different cases are shown in the Fig. 4. For pure B_40_, the geometry is relaxed by placing the two cages along the hexagonal and heptagonal rings. Our calculation for B_40_ dimers shows that they form strong covalent bonds along the heptagonal rings releasing 0.656 eV. Studies done on cluster stability of B_40_ by Yang *et al*.^25^ clearly show that they have small energy barriers of dimerization (~0.06 eV). However, dimerization through hexagonal rings is energetically not favorable. Despite these small barriers, the cages have been observed experimentally, most probably due to the presence of noble gases and the low concentration of the boron cages under experimental condition. For the decorated M_6_B_40_, the D.E. is larger than for the pure B_40_. Our investigations of the dimerization of B_40_Ti_6_ and B_40_Ca_6_ show that no barrier has to be overcome in this process. So, the dimerization will occur spontaneously.

To summarize, our investigations of the potential energy surface of the exohedrally decorated B_40_ cages reveal that the highly symmetric configurations obtained by positioning metal adatoms on high symmetry sites of the perfect borospherene cage are metastable. In global geometry optimization runs, they distort to form structures with rings of various sizes, loosing thereby symmetry. Earth alkaline and transition metals decorated B_40_ have a large energy difference between the lowest energy structure and the cage structure.

The D.E. indicate that they form strong bonds and are chemically reactive with small or no energy barriers. All these results suggest that theoretically postulated decorated structures such as B_40_M_6_ are not realizable as building blocks for applications such as hydrogen storage. More generally these findings show that structures obtained by chemical intuition are frequently not ground states and that performing unbiased global geometry optimization is essential to make reliable structure predictions.

## Methods

The scanning of potential energy surface (PES) of these decorated B_40_ cages was carried out using the Minima Hopping method (MHM)^34–38^ as implemented in BigDFT^39^ package at the density functional level of theory. The MHM is an algorithm to explore the PES of a polyatomic system in an unbiased efficient manner. The MHM consists of two parts. In the inner part short molecular dynamics trajectories are performed to cross barriers between minima followed by local geometry optimizations. In the outer part the new minimum is accepted or rejected based on energy and fingerprint difference criteria^48^.

BigDFT is massively parallel electronic structure code which uses Daubechis wavelets as basis set and gives extremely short times to solution on parallel computers. The atoms were described using soft norm conserving HGH pseudopotentials^49, 50^, with a non-linear core correction. The exchange-correlation interaction of the electrons was described through a generalized gradient approximation with the Perdew-Burke-Ernzerhof (PBE)^40^ functional. The calculations were carried out with free boundary conditions. The convergence parameters were set such that the total energy converged within 10^−5^ eV and the structure was relaxed until the maximum force component of any atom was less than 1.0 meV/Å. For the calculation of total energies with hybrid functionals PBE0^42^ and B3LYP^43–46^, the FHIaims^47, 51–54^ all electron code was also used which uses numerical atomic orbitals as basis set. The tier2 basis set was used. The scf convergence criteria set was 10^−6^ eV for total energy, 10^−6^ eV for eigenvalues and 10^−6^ for charge density. Free boundary conditions were used in the FHIaims calculations. The charge transfer of the decorated borospherene was obtained through a Bader charge analysis^55^.

For calculating the dimerization energy the structures were initially placed at a fairly large distance, where attractive interactions just start to appear, and then geometry relaxed. The distance between the two cages is defined as the distance between the centers of mass of the two cages. These calculations have been carried out using BigDFT with the PBE functional. The Libxc^56^ library was used for the calculation of the functionals.

## Electronic supplementary material

Supplemental information
