# Supplementary material for: Metastable exohedrally decorated Borospherene B40
Source: Sci Rep. 2017 Aug 8;7:7618. doi: 10.1038/s41598-017-06877-7 (PMC5548771; doi:10.1038/s41598-017-06877-7)
Supplement: Supplementary file 1 — Supplemental information [file 41598_2017_6877_MOESM1_ESM.pdf]

Supplemental Material of metastable exohedrally decorated borosphrene B_{40}

Santanu Saha[1,*], Luigi Genovese[2], and Stefan Goedecker[1]

[1]Department of Physics, Universität Basel, Klingelbergstr. 82, 4056 Basel, Switzerland

[2]Univ. Grenoble Alpes, INAC-MEM, L_Sim, F-38000 Grenoble, France

[*]santanu.saha@unibas.ch

April 11, 2017

Contents

1 Atomic coordinates of relaxed single metal placed at the center of B_{40} cage 3

1.1 Mg@B_{40}-center . . . 3
1.2 Ca@B_{40}-center . . . 4
1.3 Sr@B_{40}-center . . . 5
1.4 Li@B_{40}-center . . . 6
1.5 Na@B_{40}-center . . . 7
1.6 K@B_{40}-center . . . 8
1.7 Rb@B_{40}-center . . . 9
1.8 Sc@B_{40}-center . . . 10
1.9 Ti@B_{40}-center . . . 11

2 Atomic coordinates of relaxed single metal placed at the hexagonal hole of B_{40} cage 12

2.1 Be@B_{40}-hexagonal hole . . . 12
2.2 Mg@B_{40}-hexagonal hole . . . 13
2.3 Ca@B_{40}-hexagonal hole . . . 14
2.4 Sr@B_{40}-hexagonal hole . . . 15
2.5 Li@B_{40}-hexagonal hole . . . 16
2.6 Na@B_{40}-hexagonal hole . . . 17
2.7 K@B_{40}-hexagonal hole . . . 18
2.8 Rb@B_{40}-hexagonal hole . . . 19
2.9 Sc@B_{40}-hexagonal hole . . . 20
2.10 Ti@B_{40}-hexagonal hole . . . 21

3 Atomic coordinates of relaxed single metal placed at the heptagonal hole of B_{40} cage 22

3.1 Be@B_{40}-heptagonal hole . . . 22
3.2 Mg@B_{40}-heptagonal hole . . . 23
3.3 Ca@B_{40}-heptagonal hole . . . 24
3.4 Sr@B_{40}-heptagonal hole . . . 25
3.5 Li@B_{40}-heptagonal hole . . . 26
3.6 Na@B_{40}-heptagonal hole . . . 27
3.7 K@B_{40}-heptagonal hole . . . 28

3.8 Rb@B_{40}-heptagonal hole . . . 29
3.9 Sc@B_{40}-heptagonal hole . . . 30
3.10 Ti@B_{40}-heptagonal hole . . . 31

4 Atomic coordinates of relaxed single metal placed at the B-B bond of the hexagon ring of B_{40} cage 32
4.1 Ti@B_{40}-hexagon B-B bond . . . 32

5 Atomic coordinates of relaxed single metal placed at the B-B bond of the heptagon ring of B_{40} cage 33
5.1 Sc@B_{40}-heptagon B-B bond . . . 33
5.2 Ti@B_{40}-heptagon B-B bond . . . 34

6 Atomic coordinates of relaxed metal decorated D_{2d} B_{40} cage 35
6.1 Be_6@B_{40}-decorated cage . . . 35
6.2 Mg_6@B_{40}-decorated cage . . . 36
6.3 Ca_6@B_{40}-decorated cage . . . 37
6.4 Sr_6@B_{40}-decorated cage . . . 38
6.5 Li_6@B_{40}-decorated cage . . . 39
6.6 Na_6@B_{40}-decorated cage . . . 40
6.7 K_6@B_{40}-decorated cage . . . 41
6.8 Rb_6@B_{40}-decorated cage . . . 42
6.9 Sc_6@B_{40}-decorated cage . . . 43
6.10 Ti_6@B_{40}-decorated cage . . . 44

7 Atomic coordinates of lowest energy metal decorated M_6@B_{40} structures obtained during minima hopping runs 45
7.1 Be_6@B_{40}-lowest decorated . . . 45
7.2 Mg_6@B_{40}-lowest decorated . . . 46
7.3 Ca_6@B_{40}-lowest decorated . . . 47
7.4 Sr_6@B_{40}-lowest decorated . . . 48
7.5 Li_6@B_{40}-lowest decorated . . . 49
7.6 Na_6@B_{40}-lowest decorated . . . 50
7.7 K_6@B_{40}-lowest decorated . . . 51
7.8 Rb_6@B_{40}-lowest decorated . . . 52
7.9 Sc_6@B_{40}-lowest decorated . . . 53
7.10 Ti_6@B_{40}-lowest decorated . . . 54

8 Atomic coordinates of relaxed structures of dimers made from lowest energy metal decorated M_6@B_{40} structures 54
8.1 $(Be_6@B_{40})_2$ . . . 54
8.2 $(Mg_6@B_{40})_2$ . . . 56
8.3 $(Ca_6@B_{40})_2$ . . . 58
8.4 $(Sr_6@B_{40})_2$ . . . 60
8.5 $(Li_6@B_{40})_2$ . . . 62
8.6 $(Na_6@B_{40})_2$ . . . 64
8.7 $(K_6@B_{40})_2$ . . . 66
8.8 $(Rb_6@B_{40})_2$ . . . 68
8.9 $(Sc_6@B_{40})_2$ . . . 70
8.10 $(Ti_6@B_{40})_2$ . . . 72
8.11 $(B_{40})_2$ bonding along hexagonal ring . . . 74
8.12 $(B_{40})_2$ bonding along heptagonal ring . . . 76

The atomic coordinates of different relaxed structures obtained using BigDFT for PBE exchange correlation functional is provided. The values are given in Å.

1 Atomic coordinates of relaxed single metal placed at the center of B_{40} cage

1.1 Mg@B_{40}-center

Atom	x	y	z
Mg	2.727295	3.521467	4.228358
B	1.039010	5.394439	4.758261
B	1.087874	2.706912	1.430694
B	1.252710	5.140921	3.079916
B	4.407462	0.022685	4.761552
B	2.724903	1.020233	7.333888
B	0.105424	2.706941	6.365783
B	5.033204	1.857292	2.690196
B	5.090034	4.123718	5.626880
B	4.419171	5.391137	4.758041
B	4.364490	2.703672	1.430490
B	4.204750	5.138047	3.079697
B	0.052574	1.020050	4.001774
B	1.306895	0.334733	3.130899
B	4.140981	0.331923	3.130708
B	3.582580	0.362640	6.092917
B	1.875040	5.022423	6.075013
B	5.054736	3.575540	2.655379
B	1.865749	0.364341	6.093037
B	2.723620	0.062023	2.381666
B	0.048050	4.400058	3.976866
B	0.399542	3.580126	2.655694
B	0.417642	1.861853	2.690482
B	5.089275	1.277298	5.631435
B	0.365746	4.128367	5.627195
B	0.360823	1.282002	5.631749
B	3.596524	1.296966	1.763245
B	1.853072	1.298675	1.763349
B	1.040019	0.026030	4.761778
B	1.341370	1.827784	6.991080
B	5.347599	2.701745	6.365403
B	3.582568	5.020764	6.074898
B	3.591666	4.106807	1.746061
B	1.863543	4.108502	1.746192
B	1.345193	3.568333	7.009062
B	5.396783	1.014739	4.001417
B	2.728890	5.359867	2.398218
B	4.109985	1.825049	6.990875
B	2.728238	4.363448	7.358371
B	5.408033	4.394774	3.976504
B	4.109661	3.565607	7.008883

1.2 Ca@B_{40}-center

Atom	x	y	z
Ca	2.726413	2.726990	4.041842
B	1.049606	5.393671	4.765217
B	1.079439	2.727069	1.415540
B	1.286012	5.101301	3.114792
B	4.403203	0.060485	4.765204
B	2.726418	1.061520	7.348784
B	0.102807	2.727088	6.377623
B	5.058683	1.866630	2.661570
B	5.095180	4.153042	5.643763
B	4.403225	5.393662	4.765198
B	4.373349	2.727061	1.415522
B	4.166806	5.101295	3.114774
B	0.060885	1.059958	4.002802
B	1.285990	0.352829	3.114785
B	4.166804	0.352820	3.114767
B	3.581693	0.405755	6.092849
B	1.871135	5.048415	6.092857
B	5.058684	3.587483	2.661582
B	1.871125	0.405758	6.092861
B	2.726392	0.102338	2.404724
B	0.060889	4.394200	4.002804
B	0.394123	3.587493	2.661610
B	0.394116	1.866643	2.661598
B	5.095170	1.301107	5.643761
B	0.357652	4.153056	5.643790
B	0.357649	1.301118	5.643788
B	3.600285	1.326823	1.739999
B	1.852500	1.326829	1.740009
B	1.049602	0.060493	4.765223
B	1.336743	1.856094	7.013698
B	5.350026	2.727075	6.377595
B	3.581706	5.048409	6.092845
B	3.600293	4.127299	1.740010
B	1.852507	4.127303	1.740021
B	1.336745	3.598081	7.013693
B	5.391919	1.059943	4.002773
B	2.726405	5.351791	2.404721
B	4.116092	1.856087	7.013682
B	2.726426	4.392651	7.348775
B	5.391931	4.394187	4.002775
B	4.116099	3.598075	7.013678

1.3 Sr@B_{40}-center

Atom	x	y	z
Sr	2.728579	2.726763	4.370669
B	1.041893	5.406471	4.754872
B	1.069646	2.727036	1.419097
B	1.277854	5.115292	3.105414
B	4.411192	0.047471	4.754956
B	2.726366	1.070607	7.328160
B	0.103221	2.726973	6.341149
B	5.067698	1.865141	2.659176
B	5.112828	4.175549	5.639099
B	4.410982	5.406617	4.754871
B	4.382420	2.727017	1.418207
B	4.175380	5.115266	3.105034
B	0.046792	1.042178	3.989971
B	1.277589	0.339225	3.105390
B	4.175652	0.339237	3.105028
B	3.588358	0.387864	6.085471
B	1.864700	5.066822	6.085919
B	5.067594	3.588712	2.659151
B	1.864752	0.387628	6.085790
B	2.726499	0.102972	2.404423
B	0.046895	4.412062	3.989975
B	0.384696	3.589007	2.659048
B	0.384522	1.864830	2.659001
B	5.112905	1.278244	5.639339
B	0.339614	4.175056	5.638987
B	0.339499	1.278791	5.639179
B	3.598460	1.332726	1.750321
B	1.853869	1.332252	1.750769
B	1.041663	0.047636	4.754978
B	1.330804	1.853479	6.994826
B	5.349098	2.726972	6.340935
B	3.588421	5.066590	6.085595
B	3.598302	4.121138	1.751001
B	1.854028	4.121639	1.751315
B	1.330518	3.600800	6.994720
B	5.406096	1.042013	3.989655
B	2.726494	5.351864	2.404143
B	4.122064	1.853478	6.995426
B	2.726407	4.383533	7.327746
B	5.405980	4.412188	3.989657
B	4.122395	3.600790	6.995442

1.4 Li@B_{40}-center

Atom	x	y	z
Li	2.727343	3.543802	4.314008
B	1.024779	5.408621	4.753823
B	1.063621	2.706789	1.456857
B	1.277757	5.106005	3.113770
B	4.432388	0.002203	4.756436
B	2.724418	1.030340	7.288296
B	0.094430	2.709732	6.352510
B	5.063446	1.840949	2.681390
B	5.104093	4.131003	5.619869
B	4.434028	5.404798	4.753921
B	4.389312	2.703058	1.456953
B	4.180470	5.102752	3.113851
B	0.026934	0.998192	3.994687
B	1.308259	0.332621	3.139562
B	4.139249	0.329444	3.139643
B	3.590610	0.353135	6.076589
B	1.862653	5.060433	6.076843
B	5.077266	3.572217	2.668141
B	1.856779	0.355080	6.076539
B	2.723478	0.065321	2.388560
B	0.029090	4.411105	3.990681
B	0.377551	3.577491	2.668006
B	0.387483	1.846196	2.681254
B	5.099235	1.280676	5.611827
B	0.351808	4.136335	5.619731
B	0.350267	1.286005	5.611689
B	3.603966	1.302537	1.791173
B	1.845802	1.304509	1.791122
B	1.014291	0.006038	4.756337
B	1.331846	1.826614	6.952288
B	5.358224	2.703826	6.352660
B	3.595299	5.058490	6.076893
B	3.609186	4.104622	1.784535
B	1.846875	4.106598	1.784485
B	1.329952	3.586275	6.963538
B	5.422015	0.992137	3.994843
B	2.729395	5.338620	2.410996
B	4.118795	1.823488	6.952367
B	2.728171	4.373684	7.302774
B	5.427522	4.405049	3.990837
B	4.124638	3.583139	6.963619

1.5 Na@B_{40}-center

Atom	x	y	z
Na	2.726422	2.727100	4.372550
B	1.023155	5.423762	4.753028
B	1.061987	2.727070	1.453149
B	1.296998	5.107135	3.127423
B	4.429661	0.030380	4.753029
B	2.726408	1.062654	7.291918
B	0.091897	2.727072	6.348675
B	5.074490	1.861239	2.670115
B	5.106473	4.156481	5.617644
B	4.429660	5.423760	4.753027
B	4.390826	2.727069	1.453148
B	4.155817	5.107135	3.127422
B	0.029716	1.023817	3.992040
B	1.296999	0.347006	3.127426
B	4.155815	0.347006	3.127425
B	3.592239	0.378989	6.074953
B	1.860577	5.075154	6.074953
B	5.074490	3.592901	2.670113
B	1.860577	0.378990	6.074953
B	2.726407	0.092560	2.396394
B	0.029717	4.430324	3.992040
B	0.378324	3.592902	2.670114
B	0.378324	1.861239	2.670116
B	5.106473	1.297661	5.617644
B	0.346343	4.156481	5.617645
B	0.346343	1.297663	5.617644
B	3.606454	1.326516	1.784253
B	1.846360	1.326517	1.784254
B	1.023153	0.030380	4.753030
B	1.325854	1.847025	6.960815
B	5.360919	2.727071	6.348673
B	3.592240	5.075153	6.074953
B	3.606455	4.127623	1.784251
B	1.846360	4.127623	1.784252
B	1.325854	3.607119	6.960816
B	5.423098	1.023817	3.992039
B	2.726407	5.361580	2.396393
B	4.126962	1.847024	6.960815
B	2.726409	4.391488	7.291918
B	5.423098	4.430323	3.992038
B	4.126962	3.607118	6.960815

1.6 K@B_{40}-center

Atom	x	y	z
K	2.726411	2.727074	4.372528
B	1.019892	5.427300	4.753628
B	1.062877	2.727071	1.455835
B	1.286624	5.117663	3.121525
B	4.432924	0.026843	4.753630
B	2.726408	1.063539	7.289235
B	0.093086	2.727072	6.343250
B	5.085354	1.856851	2.663681
B	5.117000	4.166855	5.623542
B	4.432924	5.427300	4.753628
B	4.389939	2.727071	1.455835
B	4.166191	5.117663	3.121526
B	0.026179	1.020554	3.991440
B	1.286624	0.336479	3.121527
B	4.166191	0.336479	3.121528
B	3.596628	0.368126	6.081389
B	1.856189	5.086018	6.081388
B	5.085354	3.597291	2.663681
B	1.856189	0.368126	6.081388
B	2.726408	0.093749	2.401819
B	0.026180	4.433588	3.991439
B	0.367462	3.597291	2.663680
B	0.367462	1.856851	2.663681
B	5.116999	1.287288	5.623543
B	0.335817	4.166855	5.623541
B	0.335817	1.287288	5.623542
B	3.610088	1.321107	1.779835
B	1.842727	1.321108	1.779835
B	1.019891	0.026843	4.753629
B	1.320445	1.843391	6.965234
B	5.359730	2.727072	6.343250
B	3.596628	5.086018	6.081388
B	3.610089	4.133034	1.779834
B	1.842727	4.133034	1.779834
B	1.320445	3.610753	6.965234
B	5.426636	1.020555	3.991441
B	2.726408	5.360392	2.401818
B	4.132371	1.843391	6.965234
B	2.726408	4.390605	7.289235
B	5.426636	4.433587	3.991440
B	4.132371	3.610753	6.965234

1.7 Rb@B_{40}-center

Atom	x	y	z
Rb	2.726409	2.727071	4.372533
B	1.016041	5.430857	4.754728
B	1.062085	2.727071	1.456161
B	1.280057	5.125903	3.117021
B	4.436775	0.023287	4.754730
B	2.726408	1.062749	7.288909
B	0.092374	2.727073	6.342880
B	5.093755	1.853273	2.658242
B	5.125238	4.173422	5.628047
B	4.436774	5.430857	4.754729
B	4.390731	2.727070	1.456161
B	4.172759	5.125902	3.117020
B	0.022623	1.016704	3.990340
B	1.280057	0.328239	3.117022
B	4.172760	0.328239	3.117021
B	3.600205	0.359725	6.086827
B	1.852610	5.094419	6.086825
B	5.093755	3.600867	2.658243
B	1.852611	0.359725	6.086827
B	2.726408	0.093036	2.402190
B	0.022623	4.437438	3.990339
B	0.359062	3.600868	2.658243
B	0.359061	1.853274	2.658243
B	5.125239	1.280721	5.628047
B	0.327578	4.173423	5.628047
B	0.327577	1.280722	5.628047
B	3.613366	1.316416	1.774243
B	1.839451	1.316417	1.774244
B	1.016041	0.023287	4.754730
B	1.315755	1.840116	6.970825
B	5.360441	2.727072	6.342880
B	3.600204	5.094418	6.086825
B	3.613365	4.137724	1.774242
B	1.839451	4.137725	1.774243
B	1.315755	3.614029	6.970825
B	5.430193	1.016705	3.990340
B	2.726408	5.361105	2.402187
B	4.137061	1.840115	6.970825
B	2.726407	4.391396	7.288908
B	5.430193	4.437437	3.990339
B	4.137060	3.614029	6.970825

1.8 Sc@B_{40}-center

Atom	x	y	z
Sc	2.727315	2.730090	3.566000
B	1.101089	5.376426	4.789614
B	1.109049	2.738123	1.398257
B	1.360348	5.101450	3.143813
B	4.350679	0.074928	4.777281
B	2.720589	1.044075	7.381871
B	0.112062	2.722769	6.411322
B	5.008825	1.862873	2.654883
B	5.055512	4.121161	5.658417
B	4.355313	5.373613	4.794390
B	4.352332	2.735242	1.403036
B	4.100116	5.098932	3.147895
B	0.121585	1.091336	4.008159
B	1.356232	0.363159	3.128568
B	4.095860	0.360810	3.132649
B	3.575146	0.387051	6.139705
B	1.872638	5.055495	6.152251
B	5.010382	3.598376	2.660430
B	1.868505	0.388618	6.137173
B	2.726930	0.018045	2.331748
B	0.124504	4.369702	4.018735
B	0.448834	3.602451	2.653691
B	0.447324	1.866902	2.648145
B	5.053059	1.320533	5.649387
B	0.396164	4.125256	5.651645
B	0.393668	1.324714	5.642616
B	3.591871	1.314064	1.705064
B	1.866084	1.315594	1.702526
B	1.096360	0.077896	4.772506
B	1.331516	1.843654	7.052366
B	5.334904	2.718131	6.418835
B	3.579259	5.054030	6.154784
B	3.594409	4.155817	1.714079
B	1.868568	4.157353	1.711540
B	1.333086	3.595541	7.058028
B	5.329427	1.086634	4.015726
B	2.731728	5.449139	2.349339
B	4.112040	1.841188	7.056373
B	2.723546	4.390535	7.392718
B	5.332301	4.365154	4.026303
B	4.113566	3.593068	7.062035

1.9 Ti@B_{40}-center

Atom	x	y	z
Ti	2.805541	3.306347	3.533317
B	1.151675	5.403885	4.790079
B	1.153522	2.721474	1.454919
B	1.384443	5.198844	3.110201
B	4.383693	-0.026124	4.803020
B	2.714617	0.990071	7.362821
B	0.145918	2.699281	6.350731
B	4.958449	1.932095	2.740498
B	4.971522	4.097646	5.590944
B	4.274425	5.391416	4.756640
B	4.317986	2.728158	1.446321
B	4.121734	5.239499	3.066529
B	0.130701	1.037719	4.032460
B	1.432165	0.291268	3.203108
B	4.032338	0.218935	3.188789
B	3.582326	0.291668	6.162773
B	1.857953	5.069206	6.188592
B	4.975766	3.668100	2.613925
B	1.851165	0.287566	6.164832
B	2.730648	-0.082732	2.306095
B	0.198439	4.389474	4.011067
B	0.540411	3.631180	2.665963
B	0.503212	1.898919	2.735747
B	5.033958	1.252433	5.652413
B	0.442021	4.116305	5.621241
B	0.397519	1.266169	5.648769
B	3.565010	1.330940	1.895869
B	1.888423	1.296356	1.848388
B	1.053287	-0.019344	4.802604
B	1.333334	1.801967	7.023147
B	5.281296	2.693787	6.343818
B	3.549267	5.042554	6.137987
B	3.598365	4.202395	1.612847
B	1.887277	4.188499	1.633617
B	1.356369	3.562658	7.031952
B	5.285682	1.045692	4.030552
B	2.737665	5.504171	2.273904
B	4.095315	1.800320	7.018255
B	2.720463	4.385491	7.405711
B	5.260897	4.394553	3.987378
B	4.077929	3.561089	7.026080

2 Atomic coordinates of relaxed single metal placed at the hexagonal hole of B_{40} cage

2.1 Be@B_{40}-hexagonal hole

Atom	x	y	z
Be	2.720842	2.738728	-0.734394
B	0.997931	5.434482	3.250598
B	1.013685	2.737127	-0.136908
B	1.303168	5.089513	1.624645
B	4.450301	0.023918	3.234985
B	2.726305	1.046961	5.753130
B	0.094459	2.725434	4.831477
B	5.048219	1.878273	1.151904
B	5.098895	4.141006	4.081573
B	4.450170	5.434609	3.247663
B	4.428897	2.737220	-0.139780
B	4.142194	5.089618	1.622231
B	0.026321	1.022322	2.465966
B	1.303294	0.376476	1.613586
B	4.142309	0.376528	1.611165
B	3.590398	0.378998	4.533742
B	1.860034	5.073368	4.546218
B	5.048208	3.590084	1.155928
B	1.860152	0.378957	4.535227
B	2.722181	0.076715	0.874962
B	0.026208	4.439624	2.473975
B	0.396463	3.589950	1.159848
B	0.396544	1.878158	1.155828
B	5.098944	1.313644	4.074945
B	0.350688	4.140854	4.085626
B	0.350774	1.313534	4.079001
B	3.587129	1.348381	0.289670
B	1.856204	1.348335	0.291115
B	0.998065	0.023852	3.237926
B	1.336740	1.847197	5.422950
B	5.356467	2.725577	4.826936
B	3.590288	5.073425	4.544739
B	3.587079	4.123980	0.296244
B	1.856114	4.123933	0.297688
B	1.336696	3.600966	5.427049
B	5.420694	1.022428	2.461380
B	2.722046	5.392848	0.887462
B	4.115257	1.847277	5.420558
B	2.726221	4.399714	5.760976
B	5.420635	4.439814	2.469390
B	4.115215	3.601041	5.424658

2.2 Mg@B_{40}-hexagonal hole

Atom	x	y	z
Mg	2.718932	2.745437	-1.732384
B	0.992269	5.434249	3.269794
B	1.038083	2.739726	-0.079626
B	1.294140	5.097253	1.654585
B	4.456222	0.023172	3.248203
B	2.726944	1.048559	5.760116
B	0.086184	2.723354	4.855985
B	5.067296	1.873791	1.168289
B	5.107829	4.140692	4.105920
B	4.456083	5.434347	3.266124
B	4.403324	2.739824	-0.083197
B	4.150811	5.097335	1.651558
B	0.009924	1.022581	2.489211
B	1.294267	0.370774	1.638931
B	4.150927	0.370860	1.635905
B	3.593819	0.375087	4.550372
B	1.857430	5.073721	4.567771
B	5.067255	3.597571	1.174002
B	1.857550	0.375036	4.552211
B	2.721833	0.094128	0.908549
B	0.009834	4.439756	2.500530
B	0.376773	3.597434	1.178975
B	0.376819	1.873653	1.173262
B	5.107901	1.311273	4.096547
B	0.342381	4.140555	4.110967
B	0.342455	1.311131	4.101594
B	3.592164	1.350312	0.333335
B	1.850211	1.350262	0.335184
B	0.992410	0.023067	3.251874
B	1.332056	1.841536	5.435694
B	5.365689	2.723508	4.850398
B	3.593697	5.073771	4.565931
B	3.592094	4.126498	0.342547
B	1.850137	4.126447	0.344396
B	1.332011	3.601376	5.441523
B	5.437030	1.022743	2.483462
B	2.721697	5.378804	0.926062
B	4.121097	1.841618	5.432742
B	2.726858	4.392258	5.771190
B	5.436943	4.439911	2.494781
B	4.121051	3.601458	5.438571

2.3 Ca@B_{40}-hexagonal hole

Atom	x	y	z
Ca	2.718664	2.746135	-1.856035
B	1.015094	5.417929	3.272036
B	0.968280	2.740158	-0.119974
B	1.298174	5.067704	1.645254
B	4.433474	0.039376	3.249943
B	2.727077	1.043911	5.793132
B	0.093645	2.723100	4.871489
B	5.072854	1.882587	1.181790
B	5.093813	4.137123	4.119306
B	4.433319	5.418014	3.268298
B	4.472847	2.740246	-0.123808
B	4.146701	5.067776	1.642138
B	0.020535	1.032494	2.500100
B	1.298258	0.400658	1.629344
B	4.146817	0.400730	1.626228
B	3.588163	0.384348	4.562807
B	1.863237	5.063996	4.580679
B	5.072812	3.589025	1.187597
B	1.863334	0.384305	4.564693
B	2.721727	0.126327	0.881224
B	0.020484	4.429876	2.511683
B	0.371143	3.588906	1.192741
B	0.371174	1.882468	1.186933
B	5.093893	1.314452	4.109690
B	0.356529	4.137004	4.124486
B	0.356569	1.314332	4.114871
B	3.603991	1.378961	0.302353
B	1.838133	1.378915	0.304283
B	1.015170	0.039289	3.253681
B	1.337172	1.842827	5.463937
B	5.358401	2.723233	4.865733
B	3.588059	5.064040	4.578794
B	3.603929	4.098556	0.311621
B	1.838082	4.098509	0.313555
B	1.337135	3.599350	5.469938
B	5.426409	1.032630	2.494187
B	2.721616	5.347197	0.898995
B	4.116218	1.842896	5.460900
B	2.727004	4.396054	5.804588
B	5.426314	4.430012	2.505771
B	4.116180	3.599420	5.466901

2.4 Sr@B_{40}-hexagonal hole

Atom	x	y	z
Sr	2.718167	2.748120	-2.081653
B	1.015452	5.418386	3.278483
B	0.972814	2.740829	-0.110093
B	1.298797	5.069480	1.652118
B	4.433142	0.038642	3.254881
B	2.727244	1.043168	5.799012
B	0.094319	2.722564	4.876037
B	5.071516	1.881705	1.186426
B	5.093822	4.137954	4.125767
B	4.433019	5.418475	3.274566
B	4.468036	2.740924	-0.114099
B	4.145969	5.069555	1.648855
B	0.021291	1.032833	2.505328
B	1.298903	0.399360	1.635028
B	4.146075	0.399438	1.631765
B	3.588214	0.383533	4.568186
B	1.863386	5.063897	4.587287
B	5.071479	3.590628	1.192680
B	1.863491	0.383485	4.570163
B	2.721638	0.126609	0.884571
B	0.021214	4.429595	2.517758
B	0.372324	3.590502	1.198066
B	0.372364	1.881578	1.191813
B	5.093888	1.312928	4.115431
B	0.356670	4.137828	4.131195
B	0.356733	1.312800	4.120859
B	3.604365	1.377376	0.305862
B	1.837519	1.377329	0.307889
B	1.015574	0.038548	3.258797
B	1.337646	1.842304	5.469439
B	5.357957	2.722705	4.870007
B	3.588106	5.063942	4.585310
B	3.604304	4.101319	0.315836
B	1.837456	4.101272	0.317862
B	1.337607	3.598514	5.475864
B	5.425642	1.032982	2.499135
B	2.721519	5.347779	0.903676
B	4.116040	1.842379	5.466257
B	2.727168	4.395280	5.811277
B	5.425565	4.429738	2.511565
B	4.116000	3.598589	5.472682

2.5 Li@B_{40}-hexagonal hole

Atom	x	y	z
Li	2.719516	2.742932	-1.436193
B	1.012896	5.432862	3.259168
B	1.018000	2.738793	-0.066226
B	1.301342	5.102451	1.638359
B	4.435518	0.024935	3.239509
B	2.726791	1.043546	5.771726
B	0.096098	2.724021	4.846476
B	5.064343	1.867820	1.169659
B	5.091825	4.142279	4.100604
B	4.435342	5.432983	3.255741
B	4.423770	2.738913	-0.069636
B	4.143674	5.102550	1.635513
B	0.022931	1.018567	2.485072
B	1.301492	0.364969	1.624143
B	4.143830	0.365069	1.621297
B	3.590657	0.373017	4.554905
B	1.860385	5.076895	4.570758
B	5.064285	3.602597	1.174864
B	1.860537	0.372957	4.556637
B	2.721926	0.096409	0.878372
B	0.022823	4.443614	2.495351
B	0.379917	3.602431	1.179556
B	0.379972	1.867656	1.174350
B	5.091919	1.310586	4.092105
B	0.358199	4.142113	4.105343
B	0.358288	1.310419	4.096844
B	3.603437	1.345386	0.294890
B	1.839160	1.345324	0.296656
B	1.013069	0.024814	3.242936
B	1.333761	1.840783	5.438497
B	5.355510	2.724206	4.841213
B	3.590505	5.076956	4.569026
B	3.603346	4.130178	0.303242
B	1.839071	4.130116	0.305009
B	1.333705	3.603766	5.443789
B	5.424067	1.018756	2.479663
B	2.721755	5.375572	0.894208
B	4.119096	1.840881	5.435711
B	2.726682	4.399078	5.781799
B	5.423953	4.443806	2.489943
B	4.119038	3.603865	5.441003

2.6 Na@B_{40}-hexagonal hole

Atom	x	y	z
Na	2.718536	2.747006	-1.927623
B	1.009964	5.432467	3.272159
B	1.024322	2.740254	-0.051453
B	1.301525	5.100108	1.653704
B	4.438561	0.024736	3.249256
B	2.727095	1.043916	5.779237
B	0.092743	2.722866	4.861041
B	5.070678	1.868101	1.179862
B	5.095073	4.140875	4.113320
B	4.438425	5.432568	3.268376
B	4.416905	2.740361	-0.055201
B	4.143327	5.100194	1.650572
B	0.018075	1.018637	2.496489
B	1.301658	0.368423	1.636972
B	4.143454	0.368515	1.633840
B	3.591887	0.373110	4.564019
B	1.859539	5.074813	4.582561
B	5.070632	3.603898	1.186004
B	1.859671	0.373053	4.565933
B	2.721733	0.101162	0.887742
B	0.017973	4.443766	2.508601
B	0.373285	3.603746	1.191189
B	0.373335	1.867958	1.185046
B	5.095143	1.310458	4.103310
B	0.355260	4.140732	4.118542
B	0.355347	1.310304	4.108531
B	3.602560	1.351470	0.311380
B	1.839563	1.351412	0.313330
B	1.010125	0.024623	3.253039
B	1.332541	1.839211	5.448723
B	5.359315	2.723031	4.855254
B	3.591762	5.074863	4.580647
B	3.602481	4.126580	0.321201
B	1.839489	4.126528	0.323151
B	1.332491	3.602414	5.454960
B	5.428877	1.018807	2.490519
B	2.721588	5.372733	0.906383
B	4.120875	1.839300	5.445656
B	2.727003	4.395434	5.791089
B	5.428790	4.443935	2.502630
B	4.120827	3.602500	5.451891

2.7 K@B_{40}-hexagonal hole

Atom	x	y	z
K	2.717294	2.751819	-2.427345
B	1.012587	5.431173	3.286450
B	1.023572	2.741794	-0.039041
B	1.302814	5.099442	1.667167
B	4.436121	0.025342	3.260032
B	2.727544	1.041519	5.795131
B	0.094038	2.721630	4.874874
B	5.069236	1.869607	1.192597
B	5.093332	4.139892	4.127068
B	4.435988	5.431279	3.282189
B	4.416887	2.741895	-0.043260
B	4.141749	5.099527	1.663634
B	0.019302	1.018774	2.508463
B	1.302935	0.370254	1.647781
B	4.141863	0.370337	1.644248
B	3.591507	0.372874	4.576365
B	1.860473	5.072874	4.597782
B	5.069197	3.604051	1.199709
B	1.860589	0.372823	4.578519
B	2.721470	0.104375	0.893430
B	0.019214	4.443823	2.522503
B	0.374305	3.603911	1.205549
B	0.374351	1.869467	1.198437
B	5.093402	1.309793	4.115468
B	0.357425	4.139749	4.132963
B	0.357495	1.309653	4.121363
B	3.603066	1.353850	0.317996
B	1.838370	1.353796	0.320189
B	1.012722	0.025243	3.264293
B	1.334039	1.838005	5.463386
B	5.358650	2.721788	4.868317
B	3.591390	5.072927	4.595628
B	3.602999	4.126891	0.329372
B	1.838299	4.126839	0.331565
B	1.333996	3.600463	5.470608
B	5.427599	1.018932	2.501732
B	2.721340	5.371564	0.915023
B	4.120171	1.838088	5.459915
B	2.727461	4.394272	5.808870
B	5.427517	4.443988	2.515772
B	4.120127	3.600546	5.467138

2.8 Rb@B_{40}-hexagonal hole

Atom	x	y	z
Rb	2.716656	2.754219	-2.618805
B	1.012393	5.431143	3.292105
B	1.024855	2.742469	-0.033539
B	1.303065	5.099654	1.672990
B	4.436388	0.025077	3.264084
B	2.727751	1.040790	5.799655
B	0.094019	2.721075	4.880020
B	5.069144	1.869954	1.196828
B	5.093499	4.139497	4.132054
B	4.436261	5.431245	3.287617
B	4.415251	2.742571	-0.037985
B	4.141370	5.099741	1.669269
B	0.019055	1.018650	2.512796
B	1.303180	0.370513	1.652347
B	4.141492	0.370601	1.648626
B	3.591684	0.372547	4.580447
B	1.860549	5.072258	4.603177
B	5.069092	3.604404	1.204428
B	1.860666	0.372492	4.582717
B	2.721355	0.104857	0.897015
B	0.018974	4.444004	2.527737
B	0.374220	3.604260	1.210581
B	0.374256	1.869810	1.202982
B	5.093564	1.309479	4.119711
B	0.357451	4.139354	4.138260
B	0.357527	1.309330	4.125915
B	3.602783	1.354403	0.322124
B	1.838351	1.354346	0.324439
B	1.012537	0.024971	3.268570
B	1.334311	1.837376	5.468034
B	5.358960	2.721237	4.873120
B	3.591565	5.072311	4.600904
B	3.602711	4.127503	0.334275
B	1.838284	4.127449	0.336589
B	1.334264	3.599687	5.475714
B	5.427828	1.018818	2.505707
B	2.721227	5.371949	0.920023
B	4.120267	1.837460	5.464383
B	2.727664	4.393425	5.814253
B	5.427736	4.444168	2.520653
B	4.120226	3.599772	5.472063

2.9 Sc@B_{40}-hexagonal hole

Atom	x	y	z
Sc	2.695483	2.717056	-0.016746
B	1.000478	5.393839	4.750069
B	0.899600	2.711353	1.364806
B	1.284537	5.030160	3.122883
B	4.401638	0.001589	4.724193
B	2.704105	1.020229	7.261280
B	0.070086	2.691154	6.338014
B	5.055951	1.847666	2.670845
B	5.070071	4.116659	5.603524
B	4.401582	5.393925	4.746062
B	4.494621	2.711449	1.360565
B	4.113705	5.030231	3.119548
B	0.002241	0.995473	3.985612
B	1.284670	0.378364	3.103994
B	4.113771	0.378442	3.100662
B	3.570007	0.348880	6.047696
B	1.835278	5.035821	6.068709
B	5.055950	3.564581	2.677807
B	1.835384	0.348836	6.049737
B	2.698301	0.107239	2.315464
B	0.002109	4.405880	3.999442
B	0.341333	3.564458	2.683367
B	0.341404	1.847540	2.676401
B	5.070100	1.271867	5.591997
B	0.334081	4.116536	5.609104
B	0.334174	1.271744	5.597576
B	3.614515	1.348431	1.748117
B	1.780686	1.348382	1.750277
B	1.000654	0.001498	4.728200
B	1.304639	1.805905	6.937970
B	5.335858	2.691292	6.331814
B	3.569912	5.035865	6.066666
B	3.614468	4.071232	1.759207
B	1.780620	4.071184	1.761371
B	1.304599	3.571577	6.945104
B	5.398242	0.995617	3.979255
B	2.698190	5.307724	2.336635
B	4.102767	1.805977	6.934677
B	2.704034	4.354665	7.274758
B	5.398231	4.406016	3.993083
B	4.102731	3.571651	6.941810

2.10 Ti@B_{40}-hexagonal hole

Atom	x	y	z
Ti	2.678661	2.295234	0.219744
B	1.019817	5.344928	4.503765
B	0.695915	2.760756	1.124109
B	1.261372	5.002965	2.871360
B	4.374306	0.044740	4.448045
B	2.688329	1.031535	7.010531
B	0.041616	2.682596	6.109331
B	5.248134	1.828013	2.424636
B	5.073322	4.107420	5.366461
B	4.349542	5.344992	4.499037
B	4.663953	2.760833	1.118478
B	4.103369	5.003019	2.867324
B	-0.078754	1.062997	3.809164
B	1.246673	0.504960	2.877542
B	4.118264	0.505017	2.873466
B	3.551403	0.360197	5.796296
B	1.821336	5.016177	5.844043
B	5.187271	3.644438	2.360700
B	1.821838	0.360159	5.798754
B	2.681000	0.579879	1.842097
B	-0.028803	4.408052	3.759385
B	0.176092	3.644338	2.367815
B	0.115483	1.827910	2.431924
B	5.067542	1.241749	5.399530
B	0.298553	4.107325	5.373239
B	0.304545	1.241654	5.406291
B	4.112900	1.204752	1.358496
B	1.247706	1.204694	1.362565
B	0.995125	0.044671	4.452842
B	1.285661	1.806350	6.708326
B	5.332406	2.682703	6.101819
B	3.551844	5.016210	5.841586
B	3.562651	3.943279	1.562505
B	1.798425	3.943244	1.565013
B	1.268507	3.568813	6.728060
B	5.446311	1.063108	3.801318
B	2.681310	5.236497	2.125506
B	4.090100	1.806407	6.704343
B	2.688302	4.329853	7.038702
B	5.396081	4.408157	3.751685
B	4.107240	3.568869	6.724032

3 Atomic coordinates of relaxed single metal placed at the heptagonal hole of B_{40} cage

3.1 Be@B_{40}-heptagonal hole

Atom	x	y	z
Be	-0.408033	2.728523	2.825434
B	0.931923	5.519698	3.277221
B	1.024964	2.727644	-0.054014
B	1.284043	5.073358	1.685308
B	4.436891	0.014831	3.244065
B	2.704740	1.030551	5.745699
B	0.101957	2.728235	4.786600
B	5.077558	1.846269	1.167170
B	5.084277	4.121737	4.079577
B	4.440372	5.436097	3.244064
B	4.384011	2.725497	-0.008808
B	4.118525	5.109120	1.644741
B	-0.108753	0.919896	2.484353
B	1.281045	0.381593	1.685304
B	4.115473	0.342216	1.644739
B	3.583306	0.365830	4.554325
B	1.831833	5.119354	4.537360
B	5.078687	3.603835	1.167169
B	1.828769	0.334909	4.537363
B	2.693737	0.104610	0.916014
B	-0.106437	4.536812	2.484361
B	0.441482	3.558965	1.268868
B	0.440422	1.897057	1.268864
B	5.082483	1.328361	4.079579
B	0.334853	4.200695	4.093654
B	0.332972	1.255480	4.093647
B	3.596418	1.327745	0.334650
B	1.825893	1.343732	0.304694
B	0.928357	-0.064294	3.277224
B	1.341831	1.839333	5.366824
B	5.346620	2.724881	4.849440
B	3.586332	5.086193	4.554323
B	3.598208	4.124255	0.334653
B	1.827665	4.110530	0.304697
B	1.342971	3.615555	5.366820
B	5.434110	0.989903	2.480867
B	2.697090	5.348541	0.916019
B	4.084442	1.846040	5.413659
B	2.706914	4.422595	5.745695
B	5.436338	4.459746	2.480864
B	4.085572	3.605341	5.413657

3.2 Mg@B_{40}-heptagonal hole

Atom	x	y	z
Mg	-1.550413	2.729794	2.809770
B	0.962056	5.478791	3.258820
B	1.064077	2.727814	-0.026440
B	1.299010	5.070977	1.677457
B	4.476158	0.006265	3.251916
B	2.739934	1.051064	5.723090
B	0.085783	2.728565	4.843599
B	5.111494	1.846745	1.182267
B	5.135616	4.120255	4.075135
B	4.480321	5.444142	3.251913
B	4.402347	2.725261	0.014183
B	4.149496	5.109577	1.658659
B	-0.095398	0.947452	2.479269
B	1.295428	0.384296	1.677459
B	4.145847	0.341334	1.658662
B	3.623993	0.357341	4.562098
B	1.856772	5.090491	4.525975
B	5.112838	3.602689	1.182266
B	1.853157	0.363932	4.525979
B	2.722693	0.099726	0.926771
B	-0.092675	4.509943	2.479271
B	0.433133	3.574130	1.230051
B	0.431843	1.882465	1.230054
B	5.133480	1.329147	4.075137
B	0.381103	4.148104	4.075644
B	0.378934	1.308575	4.075642
B	3.621367	1.324695	0.344570
B	1.842837	1.321699	0.316156
B	0.957851	-0.023002	3.258823
B	1.344999	1.837578	5.390487
B	5.405983	2.724494	4.847039
B	3.627618	5.094372	4.562095
B	3.623510	4.127019	0.344570
B	1.844987	4.132738	0.316156
B	1.346360	3.617627	5.390487
B	5.477949	0.978802	2.481379
B	2.726711	5.353362	0.926771
B	4.134099	1.847081	5.394551
B	2.742497	4.402005	5.723087
B	5.480622	4.470072	2.481376
B	4.135443	3.603854	5.394549

3.3 Ca@B_{40}-heptagonal hole

Atom	x	y	z
Ca	-1.641180	2.730064	2.741727
B	0.993506	5.511248	3.277641
B	1.051355	2.727925	-0.010419
B	1.324821	5.092092	1.693310
B	4.477699	0.014957	3.243616
B	2.739136	1.059613	5.736182
B	0.095567	2.728638	4.801052
B	5.109706	1.847979	1.171457
B	5.135291	4.121713	4.079350
B	4.482071	5.435282	3.243680
B	4.411668	2.725213	-0.006645
B	4.163416	5.105330	1.646356
B	-0.099037	0.860971	2.470619
B	1.321005	0.363279	1.693255
B	4.159577	0.345461	1.646299
B	3.609797	0.380594	4.541825
B	1.862362	5.103315	4.542173
B	5.111119	3.601293	1.171478
B	1.858528	0.351119	4.542116
B	2.732819	0.101171	0.923023
B	-0.096023	4.596673	2.470663
B	0.432760	3.577403	1.274267
B	0.431390	1.879416	1.274247
B	5.133038	1.327451	4.079317
B	0.355289	4.188070	4.077724
B	0.352933	1.268807	4.077690
B	3.619058	1.332632	0.341300
B	1.834971	1.326079	0.331091
B	0.989014	-0.055380	3.277576
B	1.358288	1.853876	5.374927
B	5.402614	2.724356	4.849925
B	3.613581	5.071016	4.541882
B	3.621306	4.119064	0.341333
B	1.837232	4.128496	0.331123
B	1.359697	3.601348	5.374948
B	5.478264	0.986419	2.477859
B	2.737055	5.351939	0.923085
B	4.132931	1.848882	5.407773
B	2.741825	4.393374	5.736222
B	5.481067	4.462226	2.477901
B	4.134345	3.601864	5.407794

3.4 Sr@B_{40}-heptagonal hole

Atom	x	y	z
Sr	-1.869707	2.730432	2.734974
B	1.002601	5.505347	3.277400
B	1.053459	2.727991	-0.014568
B	1.328885	5.089668	1.688825
B	4.479577	0.018732	3.243970
B	2.745817	1.060273	5.742611
B	0.101209	2.728732	4.808763
B	5.113617	1.847460	1.169774
B	5.141611	4.120270	4.080592
B	4.484156	5.431354	3.244044
B	4.417227	2.725142	-0.010004
B	4.169302	5.104492	1.644864
B	-0.087744	0.867869	2.468941
B	1.324882	0.365805	1.688764
B	4.165274	0.346171	1.644800
B	3.613902	0.382968	4.544601
B	1.868709	5.098339	4.543103
B	5.115099	3.601611	1.169798
B	1.864692	0.356140	4.543036
B	2.737325	0.102683	0.922706
B	-0.084589	4.589977	2.468990
B	0.434015	3.579306	1.267643
B	0.432573	1.877694	1.267621
B	5.139251	1.328678	4.080553
B	0.369347	4.182927	4.078427
B	0.366881	1.274106	4.078388
B	3.623870	1.333890	0.339255
B	1.841120	1.327559	0.326664
B	0.997893	-0.049364	3.277325
B	1.362911	1.853200	5.385843
B	5.410795	2.724235	4.852138
B	3.617869	5.068549	4.544667
B	3.626227	4.117727	0.339290
B	1.843490	4.127078	0.326698
B	1.364391	3.602109	5.385869
B	5.481275	0.988696	2.477969
B	2.741769	5.350419	0.922773
B	4.138276	1.850247	5.409467
B	2.748638	4.392682	5.742659
B	5.484210	4.459715	2.478017
B	4.139758	3.600360	5.409493

3.5 Li@B_{40}-heptagonal hole

Atom	x	y	z
Li	-1.169705	2.729381	2.889239
B	1.018147	5.464556	3.260377
B	1.037787	2.727763	-0.045505
B	1.309194	5.087998	1.640048
B	4.440336	0.024274	3.245396
B	2.720658	1.057829	5.771246
B	0.086392	2.728478	4.840912
B	5.082730	1.851784	1.166643
B	5.097809	4.133770	4.096783
B	4.444269	5.426325	3.245376
B	4.408997	2.725309	-0.037787
B	4.145434	5.095475	1.635760
B	-0.004013	0.941503	2.474415
B	1.305760	0.367149	1.640066
B	4.141986	0.355540	1.635777
B	3.588303	0.389501	4.553631
B	1.859160	5.094444	4.568624
B	5.083999	3.597859	1.166639
B	1.855717	0.359931	4.568644
B	2.719675	0.103802	0.895997
B	-0.001415	4.515558	2.474401
B	0.390015	3.591780	1.201286
B	0.388759	1.864699	1.201291
B	5.095758	1.315887	4.096791
B	0.355057	4.162470	4.091054
B	0.352971	1.294090	4.091064
B	3.606449	1.342337	0.310046
B	1.844447	1.338017	0.290435
B	1.014167	-0.008968	3.260399
B	1.331833	1.845951	5.422110
B	5.364883	2.724635	4.856177
B	3.591704	5.062353	4.553613
B	3.608461	4.109448	0.310037
B	1.846467	4.116337	0.290424
B	1.333114	3.609197	5.422102
B	5.435391	1.005871	2.484185
B	2.723491	5.349278	0.895978
B	4.116711	1.847528	5.438834
B	2.723086	4.395298	5.771233
B	5.437892	4.443271	2.484173
B	4.117986	3.603564	5.438827

3.6 Na@B_{40}-heptagonal hole

Atom	x	y	z
Na	-1.708924	2.730092	2.864649
B	1.027703	5.457416	3.261167
B	1.052212	2.727875	-0.050430
B	1.324944	5.087167	1.642330
B	4.454181	0.024110	3.245192
B	2.736304	1.057432	5.772219
B	0.097665	2.728651	4.851968
B	5.095599	1.851273	1.166211
B	5.113365	4.132647	4.095673
B	4.458512	5.426199	3.245183
B	4.420459	2.725177	-0.036429
B	4.158508	5.095022	1.636149
B	0.011191	0.960980	2.476808
B	1.321162	0.368153	1.642336
B	4.154708	0.355757	1.636155
B	3.600235	0.389030	4.553119
B	1.868505	5.089811	4.569417
B	5.096998	3.597997	1.166212
B	1.864720	0.364656	4.569424
B	2.733118	0.101921	0.896061
B	0.014024	4.496445	2.476803
B	0.405599	3.590941	1.197675
B	0.404215	1.865849	1.197675
B	5.111106	1.316617	4.095674
B	0.371615	4.152263	4.092691
B	0.369334	1.304599	4.092693
B	3.618812	1.340429	0.309669
B	1.853078	1.334503	0.288918
B	1.023329	-0.001609	3.261175
B	1.345794	1.845814	5.431196
B	5.381318	2.724417	4.855688
B	3.603980	5.062656	4.553113
B	3.621034	4.111205	0.309668
B	1.855311	4.119962	0.288916
B	1.347206	3.609489	5.431193
B	5.449355	1.005614	2.484094
B	2.737325	5.351133	0.896056
B	4.131884	1.846896	5.437066
B	2.738979	4.395643	5.772215
B	5.452110	4.443096	2.484090
B	4.133291	3.603942	5.437064

3.7 K@B_{40}-heptagonal hole

Atom	x	y	z
K	-2.246934	2.730975	2.863253
B	1.043346	5.446657	3.259124
B	1.067349	2.727977	-0.056804
B	1.339473	5.085051	1.639316
B	4.464292	0.023852	3.245991
B	2.750590	1.055603	5.779086
B	0.110485	2.728805	4.859361
B	5.105695	1.852966	1.167546
B	5.122701	4.133270	4.096619
B	4.468973	5.426178	3.245996
B	4.433213	2.725048	-0.040817
B	4.171014	5.094043	1.635153
B	0.034021	0.981351	2.478957
B	1.335357	0.370436	1.639320
B	4.166903	0.356511	1.635153
B	3.612155	0.388184	4.554708
B	1.883059	5.084374	4.571354
B	5.107200	3.595958	1.167550
B	1.878956	0.370148	4.571339
B	2.746901	0.100891	0.891918
B	0.037086	4.476399	2.478957
B	0.417663	3.591676	1.186845
B	0.416151	1.865412	1.186845
B	5.120262	1.315624	4.096614
B	0.387676	4.146734	4.097160
B	0.385191	1.310394	4.097160
B	3.631056	1.340917	0.304624
B	1.867332	1.333433	0.283611
B	1.038594	0.009335	3.259119
B	1.361623	1.847368	5.439973
B	5.391165	2.724210	4.855829
B	3.616218	5.063325	4.554724
B	3.633465	4.110571	0.304617
B	1.869757	4.121128	0.283599
B	1.363157	3.608052	5.439982
B	5.459163	1.006949	2.484291
B	2.751469	5.352138	0.891897
B	4.145463	1.845984	5.441678
B	2.753497	4.397400	5.779106
B	5.462132	4.441351	2.484297
B	4.146992	3.604595	5.441690

3.8 Rb@B_{40}-heptagonal hole

Atom	x	y	z
Rb	-2.443319	2.731740	2.855719
B	1.048031	5.444524	3.259050
B	1.072425	2.728159	-0.058190
B	1.344700	5.084492	1.639286
B	4.468335	0.023588	3.246192
B	2.755918	1.054819	5.780514
B	0.116039	2.729073	4.861645
B	5.109693	1.852998	1.167667
B	5.127223	4.133021	4.096645
B	4.473629	5.426017	3.246227
B	4.437446	2.724852	-0.041391
B	4.175493	5.093787	1.635137
B	0.040097	0.986874	2.480051
B	1.340058	0.371282	1.639259
B	4.170846	0.356420	1.635110
B	3.616900	0.387515	4.555460
B	1.888457	5.083184	4.571495
B	5.111397	3.595371	1.167682
B	1.883830	0.371486	4.571452
B	2.751280	0.100426	0.891051
B	0.043539	4.471446	2.480073
B	0.422813	3.591829	1.185109
B	0.421109	1.865754	1.185094
B	5.124466	1.315294	4.096623
B	0.393972	4.145605	4.098688
B	0.391178	1.312005	4.098668
B	3.635519	1.340395	0.303543
B	1.872035	1.333390	0.282810
B	1.042683	0.011816	3.259011
B	1.367168	1.847465	5.442475
B	5.395806	2.723887	4.855697
B	3.621488	5.063749	4.555498
B	3.638239	4.110878	0.303554
B	1.874773	4.121352	0.282817
B	1.368900	3.608207	5.442493
B	5.463034	1.006952	2.484254
B	2.756438	5.352583	0.891065
B	4.150453	1.845627	5.442180
B	2.759201	4.398121	5.780549
B	5.466392	4.440707	2.484280
B	4.152178	3.604580	5.442200

3.9 Sc@B_{40}-heptagonal hole

Atom	x	y	z
Sc	-0.039435	2.796129	2.834452
B	2.252807	5.582937	3.276185
B	2.315242	2.793728	0.008010
B	2.588833	5.157389	1.708070
B	5.744829	0.068453	3.260135
B	3.999608	1.131324	5.713545
B	1.268037	2.794749	4.809228
B	6.379126	1.914227	1.196236
B	6.400251	4.183520	4.074687
B	5.750468	5.511798	3.260207
B	5.689736	2.790229	0.011553
B	5.410989	5.154857	1.675716
B	1.143434	0.919852	2.492597
B	2.583927	0.429461	1.708008
B	5.406086	0.426139	1.675654
B	4.886553	0.418116	4.563477
B	3.116102	5.157573	4.545303
B	6.380937	3.664767	1.196263
B	3.111197	0.428105	4.545235
B	3.991951	0.177613	0.920273
B	1.147327	4.669966	2.492648
B	1.647220	3.637510	1.268596
B	1.645467	1.951300	1.268573
B	6.397363	1.395361	4.074648
B	1.599334	4.248359	4.069055
B	1.596316	1.340480	4.069014
B	4.879635	1.411324	0.356336
B	3.081548	1.390735	0.346953
B	2.247015	0.004567	3.276112
B	2.613746	1.941400	5.341600
B	6.673553	2.789144	4.852286
B	4.891474	5.163882	4.563544
B	4.882497	4.170804	0.356368
B	3.084456	4.195121	0.346987
B	2.615511	3.645291	5.341627
B	6.759694	1.030036	2.485524
B	3.997374	5.406338	0.920336
B	5.404593	1.912474	5.399511
B	4.003052	4.452481	5.713597
B	6.763338	4.548133	2.485572
B	5.406416	3.668428	5.399541

3.10 Ti@B_{40}-heptagonal hole

Atom	x	y	z
Ti	-1.118600	2.729566	2.868741
B	0.989864	5.518577	3.260684
B	1.034060	2.727890	-0.003313
B	1.317479	5.091506	1.689649
B	4.473430	0.011302	3.261297
B	2.734555	1.062815	5.706694
B	-0.033989	2.728704	4.781130
B	5.119382	1.848559	1.193690
B	5.140215	4.105879	4.062212
B	4.477693	5.439022	3.261333
B	4.439719	2.725209	0.004785
B	4.131368	5.074465	1.681378
B	-0.102729	0.881841	2.494218
B	1.313755	0.363812	1.689622
B	4.127679	0.376423	1.681349
B	3.620484	0.343860	4.574035
B	1.835332	5.084317	4.545834
B	5.120754	3.600773	1.193705
B	1.831616	0.370147	4.545799
B	2.721417	0.129268	0.902690
B	-0.099813	4.575695	2.494244
B	0.328879	3.563385	1.243698
B	0.327558	1.893491	1.243686
B	5.138046	1.343391	4.062193
B	0.350866	4.174395	4.047314
B	0.348590	1.282424	4.047284
B	3.614838	1.358375	0.348852
B	1.806493	1.339001	0.335381
B	0.985461	-0.062768	3.260648
B	1.337041	1.881826	5.333083
B	5.418453	2.724410	4.855706
B	3.624233	5.107791	4.574072
B	3.616987	4.093334	0.348867
B	1.808676	4.115558	0.335393
B	1.338368	3.573415	5.333103
B	5.497921	0.960808	2.481612
B	2.725504	5.323846	0.902710
B	4.134518	1.852634	5.382492
B	2.737175	4.390214	5.706722
B	5.500687	4.487913	2.481636
B	4.135896	3.598198	5.382511

4 Atomic coordinates of relaxed single metal placed at the B-B bond of the hexagon ring of B_{40} cage

4.1 Ti@B_{40}-hexagon B-B bond

Atom	x	y	z
Ti	2.674250	-0.128542	0.557768
B	0.942857	5.433067	5.027559
B	0.969619	2.755444	1.719961
B	1.283406	5.079646	3.436730
B	4.398612	0.023160	4.902752
B	2.688117	0.993979	7.452635
B	0.040799	2.692689	6.597906
B	5.095571	1.851188	2.854392
B	5.069740	4.104175	5.834029
B	4.428563	5.431277	5.020834
B	4.386297	2.753694	1.713367
B	4.081521	5.078211	3.431331
B	-0.055577	1.028112	4.206552
B	1.208402	0.389613	3.303110
B	4.151203	0.388106	3.297430
B	3.560799	0.354231	6.234337
B	1.810809	5.050572	6.323844
B	5.057829	3.600382	2.926486
B	1.810088	0.355124	6.237716
B	2.678251	0.087050	2.576301
B	-0.049017	4.462502	4.250154
B	0.303642	3.602823	2.935657
B	0.263832	1.853666	2.863720
B	5.057849	1.293858	5.802960
B	0.303457	4.106619	5.843221
B	0.312349	1.296287	5.812113
B	3.885020	1.161065	1.723258
B	1.469298	1.162301	1.727920
B	0.966807	0.024913	4.909373
B	1.281062	1.786397	7.145434
B	5.333857	2.689977	6.587701
B	3.565222	5.049671	6.320457
B	3.565131	4.114159	2.080691
B	1.793591	4.115068	2.084109
B	1.278487	3.570900	7.171615
B	5.419304	1.025315	4.195991
B	2.681109	5.354606	2.658991
B	4.094786	1.784957	7.140007
B	2.689900	4.339579	7.488175
B	5.416434	4.459693	4.239614
B	4.099294	3.569456	7.166176

5 Atomic coordinates of relaxed single metal placed at the B-B bond of the heptagon ring of B_{40} cage

5.1 Sc@B_{40}-heptagon B-B bond

Atom	x	y	z
Sc	0.095024	2.627250	-0.464101
B	2.287803	5.484018	3.282600
B	2.276522	2.833734	-0.161505
B	2.561224	5.121690	1.670807
B	5.728592	0.079092	3.246992
B	4.048467	1.106329	5.794357
B	1.415065	2.791424	4.936181
B	6.360516	1.908592	1.159000
B	6.420236	4.188429	4.079779
B	5.769275	5.507749	3.259010
B	5.682337	2.800506	-0.016314
B	5.429181	5.156528	1.671712
B	1.333820	1.169402	2.579070
B	2.546393	0.497105	1.669573
B	5.391831	0.441909	1.652772
B	4.898747	0.425407	4.582760
B	3.161553	5.141374	4.576820
B	6.379011	3.674973	1.164503
B	3.160742	0.443608	4.586121
B	3.960316	0.208305	0.920258
B	1.296788	4.471357	2.537645
B	1.513225	3.723359	1.111841
B	1.459832	1.841515	1.120708
B	6.403082	1.377973	4.079943
B	1.659449	4.193289	4.147427
B	1.674246	1.373855	4.179055
B	4.853623	1.429962	0.324051
B	3.067075	1.416450	0.292291
B	2.291987	0.102760	3.286871
B	2.647877	1.901438	5.518798
B	6.683065	2.780928	4.838121
B	4.914282	5.155645	4.573423
B	4.883140	4.186421	0.341489
B	3.128043	4.170342	0.352023
B	2.653405	3.684358	5.484983
B	6.717618	1.058166	2.470883
B	3.991580	5.402167	0.960412
B	5.433713	1.903199	5.415987
B	4.055775	4.475847	5.770070
B	6.754174	4.524204	2.478914
B	5.443877	3.673871	5.413837

5.2 Ti@B_{40}-heptagon B-B bond

Atom	x	y	z
Ti	0.113595	2.791937	-0.430054
B	2.132402	5.485700	3.266133
B	2.151360	2.790413	-0.113294
B	2.357420	5.161063	1.644680
B	5.589011	0.063619	3.263534
B	3.873612	1.107733	5.769231
B	1.240682	2.791226	4.906778
B	6.184054	1.899700	1.171878
B	6.246632	4.201701	4.089537
B	5.593057	5.512276	3.263386
B	5.488094	2.787936	0.006756
B	5.243167	5.155235	1.675943
B	1.136959	1.112308	2.541867
B	2.353894	0.419557	1.644806
B	5.239650	0.421094	1.676072
B	4.742264	0.418411	4.583483
B	3.001176	5.139101	4.567864
B	6.185373	3.675199	1.171831
B	2.997687	0.440722	4.567987
B	3.802593	0.169110	0.955979
B	1.139456	4.470171	2.541780
B	1.207497	3.812269	1.048969
B	1.205972	1.770027	1.049018
B	6.244533	1.373268	4.089612
B	1.494497	4.203001	4.134787
B	1.492399	1.379036	4.134861
B	4.689538	1.394875	0.348727
B	2.927047	1.381959	0.332179
B	2.128396	0.095343	3.266276
B	2.472882	1.896218	5.469344
B	6.507592	2.787311	4.838478
B	4.745784	5.158817	4.583354
B	4.691608	4.182199	0.348651
B	2.929138	4.197737	0.332105
B	2.474209	3.684434	5.469298
B	6.565414	1.051118	2.482229
B	3.806488	5.409316	0.955834
B	5.268355	1.902007	5.418505
B	3.876111	4.470853	5.769142
B	6.567991	4.523284	2.482137
B	5.269671	3.674486	5.418459

6 Atomic coordinates of relaxed metal decorated D_{2d} B_{40} cage

6.1 $Be_6@B_{40}$-decorated cage

Atom	x	y	z
B	-0.303673	0.780618	-3.335453
B	2.811937	-1.633007	-1.297892
B	1.276399	0.441604	-2.699762
B	-0.523878	-0.625701	3.293469
B	-3.186166	-0.758053	1.183558
B	-2.124179	-1.603715	-1.868776
B	1.765072	0.738155	2.173863
B	-0.465747	3.386865	1.169674
B	-0.030611	3.254509	-0.466682
B	3.184007	0.755574	1.190654
B	1.548360	2.540795	-0.634623
B	0.031436	-3.253546	-0.473182
B	0.463644	-3.389299	1.163634
B	0.994623	-0.827198	2.441023
B	-1.768855	-0.742627	2.169300
B	-1.444651	1.224463	-2.218240
B	2.066695	1.963601	1.012182
B	-2.068501	-1.965691	1.004567
B	1.557029	-2.223171	1.642850
B	0.309472	-0.773744	-3.336472
B	1.448520	-1.219864	-2.218229
B	1.397472	-2.456327	-1.043489
B	-0.998908	0.822167	2.441037
B	-1.271707	-0.435997	-2.702955
B	-1.547167	-2.539460	-0.642501
B	2.405365	-0.791923	1.511478
B	2.428275	-1.905419	0.247120
B	-1.071960	-3.285462	0.830242
B	-2.706006	-1.167074	-0.367181
B	-1.559890	2.219726	1.644702
B	-1.395670	2.458512	-1.040897
B	2.706615	1.167767	-0.360102
B	2.528160	0.017614	-1.614267
B	-2.525253	-0.014290	-1.618628
B	0.518166	0.618894	3.295659
B	2.127585	1.607589	-1.861813
B	-2.408004	0.788790	1.508881
B	-2.809667	1.635673	-1.299407
B	1.070411	3.283703	0.838763
B	-2.428785	1.904989	0.246830
Be	0.2783931	2.136202	-2.038608
Be	0.455382	2.115826	2.163290
Be	3.566108	-0.492142	-0.071606
Be	-3.566057	0.492323	-0.076734
Be	-0.274717	-2.132039	-2.043371
Be	-0.459077	-2.120207	2.158094

6.2 Mg_6@B_{40}-decorated cage

Atom	x	y	z
B	0.421711	2.891307	-1.897597
B	-2.931909	-0.005487	-1.684994
B	-1.148837	2.287271	-1.422300
B	0.422697	-2.891217	1.897566
B	2.931988	-1.684840	0.004915
B	1.953512	-0.007755	-2.641790
B	-1.603912	-0.854984	2.291291
B	1.148400	1.422563	2.287274
B	0.422012	2.902799	1.880073
B	-2.931663	0.004755	1.685406
B	-1.148603	2.295822	1.408540
B	-0.422163	-1.897721	-2.891139
B	-1.148218	-2.296092	-1.408388
B	-1.148060	-2.287553	1.422462
B	1.604583	-2.285838	0.868567
B	1.603915	2.286238	-0.868791
B	-1.604160	0.868483	2.286105
B	1.604447	-2.291022	-0.854905
B	-1.953389	-2.641911	0.008137
B	-0.422667	1.880143	-2.902608
B	-1.604459	0.854587	-2.291060
B	-1.604255	-0.868869	-2.285876
B	1.148702	-1.408352	2.295818
B	1.148054	1.408649	-2.295985
B	1.148436	-1.422274	-2.287443
B	-2.484418	-1.370793	0.898060
B	-2.484642	-1.376296	-0.889424
B	0.422470	-2.902696	-1.880151
B	2.484662	-0.897577	-1.370655
B	1.953861	0.008245	2.641517
B	1.604010	2.291424	0.854679
B	-2.484873	1.375677	0.889767
B	-2.484915	1.370185	-0.897714
B	2.484545	0.889904	-1.376154
B	-0.421798	-1.880257	2.902682
B	-1.954062	2.641427	-0.007865
B	2.484923	-0.889277	1.375796
B	2.931581	1.685562	-0.005324
B	-0.422231	1.897618	2.891178
B	2.484637	0.898194	1.370311
Mg	0.034601	4.140780	-0.012342
Mg	-0.034768	0.012342	4.140421
Mg	-4.588111	-0.000647	0.000332
Mg	4.588057	0.000552	-0.000339
Mg	-0.035316	-0.012339	-4.140389
Mg	0.035624	-4.140731	0.012338

6.3 $Ca_6@B_{40}$-decorated cage

Atom	x	y	z
B	-2.987567	0.305838	-2.041263
B	0.000065	-3.055461	-1.678255
B	-2.257059	-1.165930	-1.319249
B	2.897746	0.553525	1.870967
B	1.773109	2.835262	-0.212195
B	0.002014	1.658576	-2.759921
B	0.876874	-1.373182	2.526218
B	-1.452626	1.301043	2.248009
B	-2.899537	0.555195	1.867738
B	-0.001915	-2.733364	2.033839
B	-2.404049	-1.097701	1.619558
B	2.021110	-0.666961	-2.947039
B	2.257851	-1.167201	-1.316705
B	2.401646	-1.099096	1.622267
B	2.346329	1.611568	0.767585
B	-2.350528	1.431880	-0.995396
B	-0.880470	-1.372657	2.525217
B	2.352446	1.430527	-0.992755
B	2.567797	-1.867922	0.166940
B	-2.018200	-0.665812	-2.949305
B	-0.874883	-1.662953	-2.247189
B	0.876435	-1.663438	-2.246195
B	1.450854	1.300206	2.249639
B	-1.445171	0.933669	-2.406004
B	1.448388	0.932842	-2.404386
B	1.289068	-2.309249	1.101450
B	1.217551	-2.344453	-0.697234
B	2.990019	0.304127	-2.037916
B	0.898848	2.269648	-1.513812
B	-0.000788	2.083605	2.474862
B	-2.346271	1.612905	0.764958
B	-1.291627	-2.308527	1.100015
B	-1.218116	-2.343775	-0.698608
B	-0.895859	2.270161	-1.514814
B	1.970073	-0.226463	3.018492
B	-2.569034	-1.866447	0.164056
B	0.888605	2.550198	1.149130
B	-1.771258	2.836274	-0.214180
B	-1.973571	-0.225324	3.016279
B	-0.888426	2.550687	1.148124
Ca	-4.357067	-0.010441	-0.012070
Ca	-0.002351	0.311552	4.364148
Ca	-0.001399	-4.499811	0.236446
Ca	0.001542	4.517031	-0.452700
Ca	0.002324	-0.417218	-4.371553
Ca	4.357075	-0.012933	-0.007189

6.4 $Sr_6@B_{40}$-decorated cage

Atom	x	y	z
B	-3.002489	0.340562	-2.044308
B	-0.000206	-3.016280	-1.749761
B	-2.239646	-1.114577	-1.320548
B	2.897237	0.531570	1.891655
B	1.768626	2.828685	-0.171527
B	0.000267	1.698985	-2.735445
B	0.895493	-1.424159	2.519201
B	-1.447680	1.269369	2.261552
B	-2.897308	0.532122	1.891431
B	-0.000338	-2.751376	1.997287
B	-2.400567	-1.125304	1.604726
B	2.034551	-0.622431	-2.955189
B	2.239535	-1.114979	-1.320361
B	2.400226	-1.125744	1.604932
B	2.354031	1.598706	0.794244
B	-2.358678	1.439785	-0.971517
B	-0.895985	-1.424004	2.519122
B	2.359026	1.439351	-0.971326
B	2.565483	-1.864220	0.139132
B	-2.034422	-0.622054	-2.955348
B	-0.883388	-1.628369	-2.287248
B	0.883271	-1.628535	-2.287188
B	1.447722	1.269098	2.261675
B	-1.443080	0.953127	-2.386558
B	1.443443	0.952852	-2.386436
B	1.269676	-2.306658	1.049146
B	1.186644	-2.301597	-0.726917
B	3.002714	0.340004	-2.044062
B	0.899986	2.271658	-1.474496
B	0.000082	2.043904	2.499346
B	-2.353811	1.599149	0.794057
B	-1.270184	-2.306434	1.049026
B	-1.187025	-2.301410	-0.727019
B	-0.899438	2.271811	-1.474554
B	1.970214	-0.276228	3.013472
B	-2.565849	-1.863756	0.138920
B	0.895974	2.532901	1.189663
B	-1.768086	2.829011	-0.171669
B	-1.970533	-0.275876	3.013309
B	-0.895596	2.533066	1.189583
Sr	-4.582946	-0.041098	-0.001500
Sr	-0.000179	0.266575	4.618491
Sr	-0.000335	-4.732980	0.148569
Sr	0.000463	4.744044	-0.424598
Sr	0.000153	-0.376330	-4.599840
Sr	4.582954	-0.041936	-0.001125

6.5 Li_6@B_{40}-decorated cage

Atom	x	y	z
B	0.420303	-3.312673	-0.580085
B	-2.918639	-0.679664	-1.605384
B	-1.169834	-2.702628	-0.410565
B	0.417511	3.312876	0.580100
B	2.918197	1.606413	-0.678312
B	1.914695	-1.012333	-2.397271
B	-1.633304	1.733009	1.857058
B	1.171367	-0.409996	2.702285
B	0.419683	-1.892610	2.779810
B	-2.919322	0.677267	1.605321
B	-1.170401	-1.589203	2.223917
B	-0.419165	0.579821	-3.312803
B	-1.171721	1.588368	-2.223884
B	-1.172231	2.701735	0.410602
B	1.631309	2.537920	-0.123374
B	1.633506	-2.536777	0.123422
B	-1.632497	0.122944	2.537350
B	1.631582	1.857560	-1.733431
B	-1.915296	2.396366	-1.013002
B	-0.417739	-2.780178	-1.892991
B	-1.631698	-1.734314	-1.857096
B	-1.632353	-0.124224	-2.537402
B	1.170149	2.224400	1.589059
B	1.171998	-2.223578	-1.589028
B	1.170827	0.410940	-2.702186
B	-2.492122	1.576121	0.295188
B	-2.491740	0.886401	-1.336650
B	0.418086	1.893015	-2.779827
B	2.491166	0.296103	-1.577041
B	1.914078	1.013878	2.397361
B	1.633162	-1.856216	1.733440
B	-2.491032	-0.888401	1.336627
B	-2.490691	-1.578092	-0.295217
B	2.491948	-1.335763	-0.887573
B	-0.420088	2.779712	1.892956
B	-1.913205	-2.397848	1.012989
B	2.490942	1.337756	0.887613
B	2.919581	-1.604089	0.678329
B	-0.418585	-0.580011	3.312664
B	2.491583	-0.294119	1.577113
Li	0.030559	-3.599684	1.521202
Li	-0.029533	1.521362	3.599901
Li	-4.274938	-0.001686	-0.000146
Li	4.274887	0.001734	-0.000018
Li	-0.028475	-1.521315	-3.599907
Li	0.027489	3.599703	-1.521115

6.6 Na_6@B_{40}-decorated cage

Atom	x	y	z
B	0.411002	1.178989	-3.118560
B	-2.921460	-1.034146	-1.387999
B	-1.174876	1.017692	-2.527828
B	0.408889	-1.178845	3.119080
B	2.922149	-1.386176	1.034957
B	1.936091	-1.565937	-2.100658
B	-1.639326	0.717782	2.422272
B	1.174698	2.527414	1.018420
B	0.409460	3.325811	-0.238488
B	-2.922930	1.032957	1.385364
B	-1.176042	2.712421	-0.254091
B	-0.408812	-3.118832	-1.179081
B	-1.175323	-2.712958	0.252870
B	-1.176439	-1.018196	2.526631
B	1.637843	-1.380278	2.117830
B	1.638873	1.380905	-2.116097
B	-1.639264	2.116634	1.379725
B	1.638493	-2.422776	0.718955
B	-1.935134	-2.102003	1.565559
B	-0.408469	-0.238676	-3.325787
B	-1.637210	-0.718426	-2.423905
B	-1.637315	-2.117185	-1.381321
B	1.174488	0.253573	2.713243
B	1.176858	-0.253101	-2.712064
B	1.176657	-2.526900	-1.017317
B	-2.508852	-0.553354	1.517083
B	-2.508117	-1.613399	0.094874
B	0.410511	-3.325516	0.238869
B	2.508978	-1.517724	-0.551666
B	1.933534	1.566743	2.102375
B	1.638242	2.423615	-0.717427
B	-2.508644	1.612396	-0.097229
B	-2.507912	0.552368	-1.519396
B	2.509094	-0.095452	-1.611733
B	-0.411437	0.238487	3.325469
B	-1.934660	2.101219	-1.567346
B	2.507673	0.096508	1.614014
B	2.922427	1.387354	-1.032312
B	-0.411150	3.118841	1.178640
B	2.507817	1.518694	0.554000
Na	0.051552	3.587607	-2.673968
Na	-0.053789	2.673976	3.587406
Na	-4.739058	-0.001264	-0.001627
Na	4.739089	0.000757	0.001712
Na	-0.049456	-2.673966	-3.587594
Na	0.051258	-3.587630	2.674145

6.7 $K_6@B_{40}$-decorated cage

Atom	x	y	z
B	-0.409536	-3.311730	-0.060326
B	2.941169	-0.907717	-1.487871
B	1.187630	-2.715229	0.006367
B	-0.412821	3.311437	0.061451
B	-2.945070	1.481950	-0.904854
B	-1.960267	-1.370682	-2.231820
B	1.654732	1.986671	1.563094
B	-1.182160	0.007627	2.717722
B	-0.405944	-1.448807	2.979060
B	2.944651	0.910884	1.479189
B	1.190473	-1.239145	2.414656
B	0.406184	0.061141	-3.312091
B	1.181921	1.240555	-2.418075
B	1.184767	2.716586	-0.009932
B	-1.655508	2.479108	-0.491356
B	-1.651392	-2.480708	0.496212
B	1.656861	0.494800	2.477476
B	-1.657204	1.564666	-1.983169
B	1.954398	2.235769	-1.372535
B	0.410559	-2.978238	-1.449400
B	1.652147	-1.984786	-1.568000
B	1.649991	-0.492888	-2.482342
B	-1.185606	2.415835	1.241649
B	-1.186814	-2.416985	-1.238197
B	-1.190252	-0.008745	-2.714123
B	2.528768	1.612230	0.049407
B	2.526951	0.685073	-1.463177
B	-0.416393	1.448577	-2.977903
B	-2.531997	0.051820	-1.607100
B	-1.954909	1.368709	2.237609
B	-1.649681	-1.566330	1.988187
B	2.532052	-0.682335	1.455803
B	2.530265	-1.609515	-0.056902
B	-2.529759	-1.460881	-0.679969
B	0.411762	2.978808	1.448090
B	1.960776	-2.233566	1.366741
B	-2.529277	1.458182	0.687526
B	-2.940737	-1.485048	0.913651
B	0.416119	-0.060502	3.311022
B	-2.527065	-0.054430	1.614648
K	-0.054739	-4.245343	2.601939
K	0.066136	2.602228	4.245211
K	5.245885	0.001319	-0.007152
K	-5.246003	-0.003967	0.007131
K	0.055344	-2.601745	-4.245438
K	-0.066407	4.245349	-2.602108

6.8 $Rb_6@B_{40}$-decorated cage

Atom	x	y	z
B	0.408163	1.095675	-3.115925
B	-2.947225	-1.073812	-1.366387
B	-1.191051	0.953354	-2.539940
B	0.411584	-1.095511	3.115620
B	2.948014	-1.367020	1.071652
B	1.963942	-1.618614	-2.065152
B	-1.657763	0.773557	2.405614
B	1.189362	2.540941	0.953148
B	0.408777	3.287256	-0.322199
B	-2.946699	1.071940	1.369027
B	-1.190588	2.694220	-0.320803
B	-0.409440	-3.116038	-1.095360
B	-1.188688	-2.695045	0.321867
B	-1.188293	-0.954237	2.541102
B	1.659836	-1.324261	2.150492
B	1.657278	1.325232	-2.152085
B	-1.658469	2.150622	1.325297
B	1.659698	-2.404553	0.773424
B	-1.963078	-2.065087	1.620249
B	-0.411168	-0.322171	-3.286813
B	-1.659194	-0.774740	-2.403940
B	-1.658476	-2.151905	-1.323853
B	1.190704	0.321554	2.693959
B	1.188653	-0.320803	-2.695089
B	1.189977	-2.540121	-0.954281
B	-2.534662	-0.513676	1.530034
B	-2.534991	-1.607922	0.135146
B	0.411068	-3.287068	0.321761
B	2.535799	-1.528091	-0.513898
B	1.964679	1.619808	2.063369
B	1.657595	2.405531	-0.774986
B	-2.536029	1.606340	-0.132793
B	-2.536249	0.512005	-1.527715
B	2.534914	-0.133061	-1.608081
B	-0.408536	0.321808	3.287246
B	-1.965805	2.063788	-1.618475
B	2.536131	0.134618	1.605800
B	2.946343	1.368804	-1.074276
B	-0.410307	3.115673	1.095705
B	2.535313	1.529678	0.511613
Rb	0.061483	4.073441	-3.195253
Rb	-0.065374	3.195441	4.073193
Rb	-5.438838	-0.000789	0.000914
Rb	5.439080	0.002045	-0.001089
Rb	-0.063993	-3.195480	-4.073102
Rb	0.066520	-4.073323	3.195264

6.9 $Sc_6@B_{40}$-decorated cage

Atom	x	y	z
B	0.783142	5.706077	3.269840
B	0.933264	2.739871	-0.010575
B	1.273579	5.116960	1.749386
B	4.672169	-0.239966	3.246824
B	2.733868	0.934720	5.722377
B	0.017793	2.723062	4.816198
B	5.084125	1.871760	1.325610
B	5.107687	4.183116	3.976903
B	4.660929	5.713397	3.265388
B	4.514630	2.746626	-0.014657
B	4.169270	5.122449	1.746106
B	-0.246331	0.791032	2.454400
B	1.282567	0.353057	1.734542
B	4.178285	0.358554	1.731177
B	3.605069	0.374265	4.375983
B	1.852807	5.084824	4.392744
B	5.080844	3.615231	1.331098
B	1.861682	0.370904	4.378029
B	2.730101	0.028761	0.893874
B	-0.253648	4.668762	2.466486
B	0.366835	3.606295	1.336479
B	0.370103	1.862872	1.331028
B	5.113130	1.287493	3.967875
B	0.343797	4.174103	3.982335
B	0.349218	1.278434	3.973290
B	3.640335	1.379873	0.432744
B	1.813754	1.376445	0.434887
B	0.794394	-0.247355	3.251285
B	1.368041	1.810936	5.276063
B	5.441082	2.733306	4.810074
B	3.596144	5.088113	4.390691
B	3.635161	4.107231	0.441292
B	1.808566	4.103753	0.443407
B	1.364619	3.637414	5.281735
B	5.707136	0.802303	2.447648
B	2.719830	5.452004	0.910798
B	4.095309	1.816058	5.272837
B	2.727087	4.516032	5.733542
B	5.699829	4.680009	2.459779
B	4.091869	3.642604	5.278549
Sc	2.719657	6.710214	2.862148
Sc	6.703190	2.741781	2.861210
Sc	2.722474	2.747344	-1.291543
Sc	2.732212	2.721368	7.006966
Sc	-1.248681	2.726778	2.870133
Sc	2.734715	-1.241571	2.837405

6.10 $Ti_6@B_{40}$-decorated cage

Atom	x	y	z
B	0.770863	5.695681	3.295474
B	0.993455	2.739957	-0.008329
B	1.271321	5.160627	1.734722
B	4.627524	-0.187725	3.260509
B	2.736682	1.014363	5.691268
B	-0.014570	2.723150	4.758050
B	5.066895	1.846648	1.277441
B	5.176587	4.202065	4.007429
B	4.616439	5.660885	3.278723
B	4.467026	2.746531	-0.011104
B	4.221918	5.222324	1.680184
B	-0.240666	0.800081	2.452450
B	1.280425	0.309463	1.719556
B	4.231220	0.259428	1.664751
B	3.632344	0.410602	4.421084
B	1.844117	5.025747	4.385388
B	5.063485	3.640567	1.283093
B	1.852795	0.430001	4.371122
B	2.735282	0.021504	0.986447
B	-0.248059	4.659927	2.464489
B	0.408954	3.610732	1.331142
B	0.412251	1.858666	1.325598
B	5.182147	1.268617	3.998307
B	0.244432	4.201627	4.025185
B	0.249772	1.250154	4.016109
B	3.637622	1.317398	0.344930
B	1.830497	1.315804	0.357079
B	0.782145	-0.237058	3.277022
B	1.309871	1.815940	5.308299
B	5.431274	2.733706	4.685525
B	3.623504	5.051550	4.435506
B	3.632310	4.170252	0.353918
B	1.825127	4.164891	0.366204
B	1.306487	3.631879	5.313832
B	5.682641	0.815681	2.409077
B	2.725053	5.458736	1.003314
B	4.183317	1.840679	5.453649
B	2.730221	4.436519	5.701986
B	5.675373	4.666847	2.421074
B	4.179961	3.617176	5.459210
Ti	2.709612	6.442807	2.925141
Ti	6.434691	2.741455	2.808208
Ti	2.716145	2.746728	-1.133671
Ti	2.718365	2.721780	6.865919
Ti	-0.988850	2.727503	2.787090
Ti	2.723629	-0.974596	2.901991

7 Atomic coordinates of lowest energy metal decorated $M_6@B_{40}$ structures obtained during minima hopping runs

7.1 $Be_6@B_{40}$-lowest decorated

Atom	x	y	z
B	-1.064643	-2.537136	-1.435147
B	-2.873919	0.188001	1.318497
B	-2.036976	-2.355151	0.056335
B	1.123736	3.047831	0.034537
B	1.530213	2.182723	-1.320574
B	-0.680701	-0.517089	-3.369987
B	0.019922	0.865450	3.745192
B	1.989851	-0.836884	1.715602
B	1.487554	-2.500587	1.258638
B	1.255397	-0.084185	3.041450
B	0.097363	-3.016428	1.961464
B	-2.082995	1.916297	-1.405303
B	-1.870739	2.546651	0.072314
B	-0.259819	3.251010	0.872072
B	2.311056	0.606520	-1.185522
B	0.504895	-2.981669	-1.587704
B	-0.322760	-0.665510	3.585477
B	0.536694	2.329408	-2.690356
B	-1.716127	3.152841	1.567762
B	-2.592488	-1.785203	-1.380350
B	-2.708927	-0.744886	-0.040388
B	-2.524458	0.934920	-0.177993
B	2.056490	1.579195	0.194504
B	-1.719114	-1.507205	-2.685803
B	0.054562	0.939287	-3.452634
B	1.156880	1.682065	3.025345
B	-2.414028	1.714498	1.531352
B	-1.014469	1.921122	-2.642776
B	0.977232	-0.505906	-3.176906
B	2.738728	-1.767655	0.466676
B	1.589166	-2.435505	-0.504411
B	-0.987835	-1.844864	2.700777
B	-2.121962	-1.279292	1.479486
B	0.038836	-1.892472	-2.683929
B	1.204973	2.340181	1.541645
B	-1.470111	-2.868766	1.492020
B	2.713791	-0.014102	0.413125
B	2.453719	-0.476884	-2.437143
B	0.713617	-1.548179	2.505783
B	2.747774	-1.117824	-1.007490
Be	-0.119930	-3.178842	0.098757
Be	2.460620	0.960869	1.923876
Be	-0.549209	2.171484	2.511666
Be	1.876067	1.151751	-2.924218
Be	-2.129995	0.105075	-1.856684
Be	-0.377935	2.875047	-1.149034

7.2 $Mg_6@B_{40}$-lowest decorated

Atom	x	y	z
B	-0.806710	-2.506360	1.469350
B	3.149548	-1.977767	-0.317550
B	0.406047	-2.690379	0.257534
B	-0.484249	2.572636	-0.944991
B	-0.497386	2.816344	2.401242
B	0.422809	-0.019171	3.435703
B	-0.432846	1.463384	-3.718114
B	-2.904225	0.712414	-0.258854
B	-2.725321	-0.525400	-1.318129
B	0.277703	0.067781	-4.181593
B	-0.683763	-2.397253	-2.646700
B	3.019824	0.333161	1.955985
B	3.074894	0.866580	0.316273
B	2.109135	1.812461	-2.441865
B	-0.344186	2.738974	0.696095
B	-2.482086	-2.348423	1.204945
B	-1.314691	0.119206	-3.830521
B	1.032834	2.448435	1.953693
B	3.028832	0.988181	-1.342833
B	0.775707	-2.527962	1.890248
B	2.029366	-2.224762	0.894931
B	3.147507	-0.905748	0.924478
B	-1.828026	2.202557	-0.083817
B	-0.198495	-1.473559	2.875755
B	1.619022	0.857993	2.681424
B	1.226058	1.280735	-3.619211
B	3.439086	-0.424101	-0.757630
B	2.503513	1.819788	1.571777
B	0.202684	1.555721	3.214924
B	-3.056648	1.816606	1.040820
B	-3.071851	-0.970547	0.215493
B	-0.511942	-1.334918	-3.775919
B	1.790823	-2.725849	-0.805511
B	-1.838598	-1.670394	2.564449
B	0.438954	2.217390	-2.343545
B	0.352181	-2.814875	-1.424917
B	-1.849310	2.745498	1.551462
B	-3.212605	-1.063911	1.957451
B	-1.811828	-1.081684	-2.641252
B	-3.625076	0.332055	1.267779
Mg	-1.978964	-2.788056	-0.898883
Mg	-2.179566	1.565627	-2.253590
Mg	1.594457	-0.836151	-2.314370
Mg	-1.828697	0.663441	2.848762
Mg	2.276665	-1.447519	3.155746
Mg	1.749420	2.757821	-0.426522

7.3 Ca_6@B_{40}-lowest decorated

Atom	x	y	z
B	-1.548978	-0.612909	-2.556742
B	-4.118903	-1.064418	0.835883
B	-2.665630	0.047399	-1.414638
B	3.352502	-0.271536	1.781862
B	2.493610	-2.809462	0.348567
B	0.408246	-2.795819	-1.922426
B	1.113479	1.601214	2.259279
B	1.986454	1.828677	-1.471481
B	0.403260	2.410694	-1.455586
B	-0.303239	2.267326	1.639838
B	-2.152690	3.092716	-0.395135
B	-1.250461	-1.638860	2.235539
B	-0.416768	-0.588486	3.322810
B	2.340023	0.564523	2.756432
B	3.371150	-1.490160	0.751285
B	-0.344586	0.095777	-3.346465
B	-1.026294	3.512738	0.759120
B	0.978604	-3.275556	0.842277
B	0.887537	0.363076	3.399527
B	-2.645246	-1.591466	-1.851770
B	-3.695939	-1.146272	-0.701124
B	-2.971579	-0.747531	1.868118
B	3.103711	1.779415	-0.142082
B	-1.138173	-2.330491	-1.799590
B	-0.245918	-2.933228	-0.314225
B	-0.525484	1.005660	2.754391
B	-1.851054	-0.150367	2.810351
B	-0.479681	-2.786392	1.355833
B	1.417728	-3.285516	-0.750475
B	3.341045	0.822068	-1.391268
B	0.821817	1.325469	-2.675438
B	-0.789201	1.971002	-0.114686
B	-3.374563	0.236355	0.206016
B	0.122135	-1.360052	-2.644976
B	2.745523	1.303988	1.350453
B	-2.510808	1.455453	-0.522998
B	3.413628	0.087084	0.136232
B	1.206447	-0.226422	-3.266460
B	-0.634624	3.592712	-0.869500
B	2.407948	0.720978	-2.717580
Ca	-1.740627	2.191904	-2.796364
Ca	1.526836	3.631623	0.547887
Ca	-2.875150	2.076882	1.909547
Ca	2.903927	-1.718329	-1.934158
Ca	-2.756127	-3.155707	0.408874
Ca	1.716114	-2.005755	2.775040

7.4 $Sr_6@B_{40}$-lowest decorated

Atom	x	y	z
B	0.645895	3.130061	-2.394934
B	0.909586	-1.097466	-3.598103
B	-1.216692	0.720512	-2.839862
B	0.649858	-2.515280	2.690606
B	2.041401	0.023578	2.741362
B	2.267306	1.570026	-0.147075
B	-1.647047	-1.787368	1.231325
B	-1.159020	2.440756	2.496410
B	-2.159637	2.342986	1.156482
B	-2.447253	-2.277414	-0.135800
B	-2.295146	0.404268	-1.428048
B	2.955767	-1.286170	-1.228402
B	1.583765	-2.241897	-1.019837
B	-0.606272	-3.035510	1.829530
B	0.901132	-1.079771	3.359527
B	0.664015	4.289379	-1.360759
B	-2.562118	-0.663195	0.259533
B	1.957160	-1.475345	2.032659
B	0.146716	-3.013597	-0.969868
B	-0.054098	1.876626	-3.159749
B	0.211758	0.391832	-3.674721
B	2.026766	-1.535373	-2.528883
B	-0.605296	-0.254004	3.694962
B	1.270179	2.780169	-0.685874
B	2.893795	0.080180	-0.348420
B	-1.091006	-3.225353	0.254733
B	0.409140	-2.098567	-2.316739
B	2.365201	-1.361553	0.360072
B	2.031136	0.323716	1.077393
B	-0.532202	1.358106	3.616396
B	-0.771143	3.396543	1.221990
B	-2.798038	-1.223170	-1.307505
B	-0.709707	-0.977532	-3.279295
B	0.728153	1.250054	0.311527
B	-0.859487	-1.616786	2.728263
B	-2.211938	-0.686750	-2.690067
B	0.875415	0.543822	3.804690
B	0.228845	4.046366	0.108508
B	-2.377839	1.043033	0.139951
B	-0.759009	1.553680	0.789920
Sr	-1.921310	3.180193	-1.509177
Sr	-2.937733	0.258805	2.738424
Sr	-1.922601	-3.412220	-2.536821
Sr	1.602597	2.778855	2.457604
Sr	2.581185	1.163095	-2.830737
Sr	1.697824	-4.082324	0.888807

7.5 $Li_6@B_{40}$-lowest decorated

Atom	x	y	z
B	2.962004	-0.514783	0.962214
B	1.522572	-0.411891	-2.938972
B	2.831507	-1.493312	-0.422211
B	-3.070384	1.106421	-0.570487
B	-1.349804	2.665682	1.732237
B	1.867699	2.599283	1.038785
B	-2.337626	-1.490018	-1.539731
B	-1.085558	-1.680464	2.287394
B	0.324133	-2.638190	2.337438
B	-1.544049	-2.928053	-1.382913
B	0.480536	-3.114776	0.632363
B	1.053935	2.226536	-1.839836
B	-0.420482	2.228905	-2.663012
B	-2.387799	0.174537	-1.944780
B	-2.293793	1.994136	0.532150
B	1.898361	-0.093653	2.126720
B	-1.108831	-3.526404	0.040022
B	-0.868560	2.923752	0.160411
B	-1.189735	0.753208	-2.974558
B	2.598218	0.182338	-0.488117
B	2.019493	0.887692	-1.924745
B	0.461483	0.838063	-2.712019
B	-2.840321	0.304089	0.855195
B	2.562120	1.082640	1.018651
B	0.865491	3.083945	-0.172565
B	-0.783395	-1.911350	-2.349716
B	-0.112941	-0.664579	-3.200385
B	-0.259682	3.190950	-1.342126
B	0.273611	2.691491	1.448011
B	-2.176069	-0.484719	2.127017
B	0.701643	-1.151819	2.823138
B	0.133797	-2.895906	-1.043246
B	2.693698	-0.708901	-1.887577
B	1.334144	1.584882	2.214005
B	-3.150465	-0.479064	-0.643478
B	1.606033	-2.658829	-0.439708
B	-1.817957	1.113166	2.013129
B	0.787586	0.422971	3.244008
B	-0.953112	-3.207217	1.601585
B	-0.747769	-0.051358	2.799926
Li	2.460272	-2.534524	1.621742
Li	-2.830744	-2.247059	0.646504
Li	1.609770	-2.544188	-2.723504
Li	-0.420141	2.215928	3.635618
Li	3.064184	2.490075	-0.820150
Li	-2.363074	2.670370	-1.874426

7.6 $Na_6@B_{40}$-lowest decorated

Atom	x	y	z
B	0.312000	-2.859782	0.905664
B	-2.899777	-1.450188	-1.449078
B	-1.313448	-2.663015	0.724504
B	0.612582	2.536059	-0.732104
B	2.760438	0.848059	-1.325727
B	2.386951	-1.810477	-0.705979
B	-1.853908	1.993058	0.727481
B	1.427907	1.550455	2.512447
B	0.346581	0.791035	3.517393
B	-3.245679	1.134737	0.764684
B	-2.127966	-0.637675	2.752634
B	-0.363303	-0.725603	-3.502365
B	-1.448547	0.377456	-3.117773
B	-1.126388	2.288484	-0.914100
B	1.829075	2.146791	-1.784331
B	1.821579	-2.217042	1.052640
B	-2.279366	1.022999	2.124410
B	1.889965	0.852519	-2.768459
B	-1.948288	1.609287	-2.179634
B	-0.344904	-3.179997	-0.544388
B	-1.599755	-2.377724	-1.123324
B	-1.583258	-1.282060	-2.511800
B	1.044277	2.405347	1.030781
B	1.311824	-2.944229	-0.371016
B	2.351245	-0.722259	-2.099924
B	-2.674758	1.404416	-0.741301
B	-2.558153	0.091355	-1.882415
B	1.252716	-0.495648	-3.333198
B	3.418576	-0.551274	-0.787578
B	2.581051	1.963552	1.374576
B	1.616246	-0.105006	3.144581
B	-2.971655	-0.408958	1.279046
B	-2.725141	-1.701880	0.166801
B	2.995959	-0.888090	0.765742
B	-0.450131	2.786609	0.513742
B	-2.381957	-1.877245	1.744429
B	2.276099	2.108785	-0.223119
B	2.401513	-1.196781	2.245190
B	-1.257924	0.562670	3.349423
B	2.644930	0.390001	1.866952
Na	-0.079219	-1.861308	3.285258
Na	-0.742979	2.993036	3.026600
Na	-4.890225	-0.231837	-0.569116
Na	4.773952	1.142764	0.442762
Na	0.704919	-2.862497	-2.847243
Na	0.106342	2.051104	-3.803767

7.7 K_6@B_{40}-lowest decorated

Atom	x	y	z
B	6.069500	2.986269	4.193894
B	4.646286	3.094899	0.301557
B	5.996627	2.024744	2.800621
B	0.153611	4.659765	2.633990
B	1.791189	6.239202	4.974832
B	5.007123	6.123915	4.274842
B	0.824805	2.043838	1.680538
B	2.036261	1.881046	5.509562
B	3.454284	0.922375	5.556471
B	1.601297	0.589049	1.778009
B	3.609004	0.455541	3.850788
B	4.174222	5.738120	1.418877
B	2.676314	5.735339	0.599519
B	0.773047	3.711698	1.259020
B	0.854233	5.551240	3.770249
B	5.037483	3.436541	5.369594
B	2.007873	0.089098	3.251429
B	2.272610	6.494400	3.392496
B	1.947208	4.272440	0.189578
B	5.718817	3.693431	2.746573
B	5.136817	4.409773	1.316865
B	3.588414	4.350450	0.540554
B	0.337402	3.868814	4.080121
B	5.678065	4.599292	4.243714
B	4.006209	6.584231	3.057522
B	2.369176	1.612302	0.825601
B	3.019117	2.870531	-0.038166
B	2.878225	6.736917	1.879165
B	3.409445	6.228350	4.676150
B	0.961399	3.090154	5.380016
B	3.834918	2.394464	6.071980
B	3.275568	0.673798	2.180644
B	5.838088	2.807177	1.340130
B	4.483891	5.125924	5.465818
B	0.054390	3.066990	2.592549
B	4.755655	0.858904	2.783127
B	1.330458	4.689669	5.267859
B	3.928266	3.974328	6.491244
B	2.170705	0.350027	4.828134
B	2.395682	3.506373	6.051322
K	6.251168	0.339673	5.278536
K	-0.636251	0.774157	4.111148
K	4.996022	0.344929	-0.154604
K	2.674387	6.281841	7.683728
K	6.982088	6.439861	2.167722
K	0.107351	6.821346	0.848486

7.8 $Rb_6@B_{40}$-lowest decorated

Atom	x	y	z
B	6.419715	2.969090	4.200291
B	4.983674	3.065987	0.310377
B	6.348223	2.004788	2.809614
B	0.516029	4.651491	2.653112
B	2.153792	6.241763	4.986029
B	5.366337	6.112039	4.271342
B	1.175596	2.029899	1.708665
B	2.388288	1.885560	5.533874
B	3.805569	0.924483	5.573041
B	1.943705	0.567648	1.791393
B	3.952360	0.454954	3.868507
B	4.515188	5.712470	1.427576
B	3.011672	5.708737	0.612546
B	1.119913	3.696961	1.280483
B	1.211997	5.546989	3.788665
B	5.394149	3.429358	5.378017
B	2.347658	0.090687	3.274109
B	2.627828	6.490523	3.400650
B	2.286335	4.246900	0.196467
B	6.065839	3.672130	2.751806
B	5.476465	4.383418	1.322730
B	3.927524	4.323712	0.551782
B	0.694309	3.870962	4.105733
B	6.031806	4.584248	4.243618
B	4.360231	6.565325	3.055996
B	2.709900	1.587961	0.836737
B	3.354786	2.846342	-0.031812
B	3.226511	6.720474	1.881103
B	3.770541	6.222370	4.678377
B	1.315800	3.096728	5.410574
B	4.189929	2.395166	6.089562
B	3.617797	0.663061	2.199790
B	6.178973	2.779125	1.347199
B	4.847797	5.121745	5.470466
B	0.415297	3.057163	2.624088
B	5.100035	0.846250	2.796972
B	1.690942	4.694415	5.288996
B	4.290746	3.976449	6.501966
B	2.517082	0.353871	4.850831
B	2.755056	3.510484	6.072653
Rb	6.715338	0.207451	5.373841
Rb	-0.463197	0.683334	4.185926
Rb	5.374946	0.190880	-0.261819
Rb	3.039189	6.392006	7.845128
Rb	7.480539	6.494187	2.101731
Rb	0.324837	6.906745	0.765585

7.9 $Sc_6@B_{40}$-lowest decorated

Atom	x	y	z
B	-0.511040	3.916963	-0.175685
B	0.537151	1.099499	3.049661
B	-1.029073	3.492958	1.273334
B	0.135610	-3.644208	-0.689172
B	2.108408	-1.593775	-1.931051
B	1.213616	2.457320	-2.010952
B	-0.905744	-2.925783	1.879352
B	-1.875611	-0.770392	-2.655443
B	-2.395846	0.569125	-1.705833
B	-2.035391	-1.738919	2.045773
B	-2.678702	1.008143	1.395630
B	2.541568	0.898664	1.707179
B	2.871212	-0.683262	1.928649
B	0.287033	-3.660199	0.959111
B	1.166888	-2.862401	-1.759023
B	-0.486814	2.951794	-1.559388
B	-2.896023	-0.648043	1.163647
B	2.762313	-0.169666	-1.132958
B	1.809981	-1.688834	2.684118
B	0.640470	3.211755	0.885814
B	-0.199555	2.304338	2.173251
B	1.438424	2.139352	1.897453
B	-1.593071	-3.532737	-1.172026
B	0.926252	3.564271	-0.808921
B	2.262147	1.189855	-2.015178
B	0.769797	-2.812018	2.237849
B	1.725337	-0.069190	2.963255
B	3.183145	-0.003635	0.472788
B	2.109219	-0.214330	-2.769454
B	-1.700588	-2.391661	-2.397198
B	-1.632822	2.003665	-2.268766
B	-2.091226	-0.200989	2.545217
B	-1.148029	1.103433	3.004472
B	0.906131	0.848973	-3.062872
B	-1.223961	-3.789761	0.367745
B	-1.911692	2.361925	2.036544
B	-0.346408	-3.299884	-2.276702
B	-0.194079	2.063528	-3.000822
B	-2.886525	0.350892	-0.131097
B	-0.877145	0.513200	-2.835633
Sc	-2.578713	2.598954	-0.275384
Sc	-1.170146	-1.398008	-0.311867
Sc	-0.280651	-1.080498	3.536194
Sc	0.239619	-1.455664	-3.484826
Sc	2.726903	2.275760	-0.126495
Sc	2.287629	-2.290507	0.349714

7.10 $Ti_6@B_{40}$-lowest decorated

Atom	x	y	z
B	2.384427	-2.236109	1.163124
B	0.053163	0.031538	4.045802
B	2.498835	-0.927835	2.076579
B	-1.784856	1.905604	-1.488400
B	-2.605403	-0.644548	-2.591724
B	0.010987	-2.870879	-0.354788
B	0.616810	3.333911	-0.755154
B	1.154967	0.100101	-3.514922
B	2.302878	-0.051850	-2.220021
B	0.546406	2.970145	0.885536
B	2.976822	1.422594	0.464566
B	-2.584883	-0.887144	2.112973
B	-2.578109	0.485789	2.940725
B	-0.914240	2.610489	-0.190501
B	-2.770917	0.627761	-1.565124
B	1.751854	-2.377001	-0.373796
B	1.887354	2.391171	-0.345717
B	-2.802109	-0.943289	-0.914537
B	-1.721423	1.877458	2.662919
B	1.158370	-2.049263	2.332244
B	0.082970	-1.489858	3.433369
B	-1.386843	-0.723152	3.269704
B	-0.865794	2.180713	-3.016214
B	0.904019	-3.006325	0.979569
B	-1.726562	-2.320787	-0.434001
B	-0.998721	2.581037	1.443144
B	-1.271208	0.934977	3.903076
B	-2.636176	-1.469543	0.624912
B	-2.196030	-2.098201	-2.084840
B	-0.226493	0.888221	-3.714503
B	2.386736	-1.747039	-1.753175
B	2.019071	2.287106	1.426178
B	1.300469	-0.194704	2.988543
B	-0.821313	-2.836362	-1.791382
B	-0.631356	3.121177	-1.772416
B	2.228702	0.688238	1.889543
B	-1.701358	0.668834	-2.940771
B	0.752865	-2.364076	-1.917259
B	2.718852	1.110431	-1.119059
B	1.496517	-1.404917	-3.062898
Ti	3.265890	-0.757135	-0.097558
Ti	1.278640	2.129967	-2.525536
Ti	0.487016	1.934871	2.915505
Ti	-0.633520	-1.311527	-3.481233
Ti	-1.004736	-2.481620	1.801915
Ti	-2.402569	0.911031	0.665603

8 Atomic coordinates of relaxed structures of dimers made from lowest energy metal decorated $M_6@B_{40}$ structures

8.1 $(Be_6@B_{40})_2$

Atom	x	y	z
B	-1.395655	-2.434406	-1.671443
B	5.774393	-2.384822	-1.160331
B	-3.091425	-0.118532	1.491802
B	4.170159	0.401115	1.179732
B	-2.331621	-2.483618	-0.129403
B	4.440705	-2.330072	0.291103
B	0.826438	3.056661	0.482852
B	8.157153	2.798815	-0.076064
B	1.275544	2.295993	-0.961172
B	8.837825	1.906741	-1.354419
B	-1.073672	-0.154145	-3.285396
B	6.699663	-0.838778	-3.497888
B	-0.037254	0.309698	3.856444
B	6.896104	1.417895	3.423701
B	1.790794	-1.161315	1.502323
B	8.937838	-0.767605	1.994802
B	1.214582	-2.710010	0.872308
B	8.296397	-2.416788	1.695582

Atom	x	y	z
B	1.118018	-0.523280	2.950295
B	8.100511	0.266701	3.004438
B	-0.136110	-3.317675	1.582804
B	6.820052	-2.717876	2.311141
B	-2.402527	1.987821	-0.986482
B	5.201978	1.995740	-2.269462
B	-2.122957	2.401181	0.563373
B	5.231838	2.638112	-0.674109
B	-0.537715	3.028699	1.357834
B	6.553803	3.306063	0.089149
B	2.422685	0.827378	-1.086773
B	9.644304	0.350673	-1.036333
B	0.194777	-2.831640	-1.949019
B	7.317711	-2.944494	-1.131565
B	-0.454737	-1.160134	3.472739
B	6.351564	-0.074255	3.408133
B	0.205267	2.553842	-2.246533
B	8.080759	2.007569	-2.891675
B	-1.938130	2.791736	2.131606
B	5.270713	3.055602	0.959795
B	-2.922641	-1.698414	-1.442999
B	4.399952	-1.437454	-1.343686
B	-2.984089	-0.846650	0.007513
B	4.119409	0.068112	-0.469407
B	-2.782842	0.842790	0.109938
B	4.058507	1.598484	-1.264528
B	2.245121	2.184881	0.512546
B	9.317237	1.490251	0.204162
B	-2.087477	-1.251827	-2.733089
B	5.488311	-1.340891	-2.548432
B	-0.329090	1.295214	-3.186361
B	7.497395	0.584293	-3.535773
B	1.069070	1.206611	3.186439
B	8.263101	1.987420	2.873303
B	-2.607762	1.365625	1.902891
B	3.973624	2.133329	0.701270
B	-1.365475	2.159954	-2.236813
B	6.533040	1.892079	-3.201041
B	0.569045	-0.187613	-3.120199
B	8.271142	-0.922689	-3.064860
B	2.714641	-2.144060	0.232718
B	9.623214	-1.917844	0.872372
B	1.377835	-2.505206	-0.864935
B	8.475114	-2.594194	-0.054462
B	-1.148436	-2.260743	2.505469
B	5.867492	-1.584756	3.111573
B	-2.379054	-1.597340	1.430355
B	5.084336	-0.571358	2.082926
B	-0.367620	-1.642794	-2.879364
B	7.073419	-2.027295	-2.434337
B	1.071644	2.139163	1.839546
B	8.771477	2.465878	1.442588
B	-1.720598	-3.155483	1.213069
B	5.336190	-2.320175	1.710413
B	2.675598	-0.313106	0.394846
B	9.860311	-0.228773	0.643949
B	2.096806	-0.237634	-2.550958
B	9.652622	-0.888618	-2.144587
B	0.499878	-1.895736	2.214388
B	7.571561	-1.279703	2.793745
B	2.658623	-1.275024	-1.386187
B	9.762653	-1.426598	-0.645360
Be	-0.353199	-3.192211	-0.263897
Be	6.601917	-2.965406	0.485916
Be	2.410806	0.678283	1.947213
Be	9.632646	0.956430	2.011160
Be	-0.669670	1.757953	2.847023
Be	6.912824	2.724693	1.889388
Be	1.522622	1.416326	-2.706985
Be	9.285603	0.698478	-2.816210
Be	-2.465941	0.248419	-1.680260
Be	4.046703	0.111703	-2.517303
Be	-0.677398	2.897270	-0.653629
Be	6.960028	2.777367	-1.634944

8.2 $(Mg_6@B_{40})_2$

Atom	x	y	z
B	-0.689016	-2.203194	1.731647
B	7.091656	-5.787658	1.159492
B	3.583207	-2.577420	-0.230945
B	10.821324	-4.968210	-0.989596
B	0.657815	-2.442939	0.692187
B	8.141455	-5.760120	-0.241516
B	-0.584027	2.357874	-1.440547
B	7.435813	-0.310061	-0.351436
B	-0.844140	3.206723	1.795808
B	7.823071	-0.633436	2.952675
B	0.123434	0.619667	3.343329
B	8.646804	-3.672131	3.333716
B	-0.346574	0.909851	-4.036302
B	6.995791	-0.865161	-3.119052
B	-3.000559	0.525570	-0.606050
B	5.113401	-2.229801	0.279952
B	-2.700762	-0.837544	-1.461958
B	4.895561	-3.394877	-1.014514
B	0.388802	-0.503758	-4.358406
B	7.565921	-2.153026	-3.937373
B	-0.386140	-2.602914	-2.306533
B	6.723165	-4.921292	-2.904263
B	2.808532	0.939210	2.094381
B	11.097885	-3.115271	1.644415
B	2.923414	0.886537	0.416166
B	10.985364	-2.272913	0.133566
B	2.100999	1.252794	-2.536879
B	9.760202	-0.779793	-2.278029
B	-0.568857	2.813133	0.152339
B	7.764604	-0.509263	1.248522
B	-2.347346	-2.193280	1.331482
B	5.404276	-5.567217	1.160018
B	-1.198897	-0.462381	-4.018315
B	6.059547	-2.165131	-3.304073
B	0.708816	2.834102	1.493725
B	9.271713	-0.956226	2.310801
B	2.942865	0.604940	-1.239602
B	10.737555	-1.848338	-1.471006
B	0.823698	-2.214748	2.343776
B	8.708306	-5.888720	1.349235
B	2.335192	-2.076660	1.481032
B	9.854913	-5.431783	0.273046
B	3.361674	-0.507193	1.516388
B	11.043737	-4.157538	0.401926
B	-1.971660	2.088281	-0.613038
B	6.246949	-0.951128	0.544476
B	-0.262268	-1.009464	3.044033
B	7.915130	-4.990790	2.599770
B	1.314412	1.468345	2.551684
B	9.798680	-2.709068	2.603285
B	1.310050	0.754291	-3.804396
B	8.655576	-1.091394	-3.351294
B	3.051767	-0.757979	-0.282795
B	11.102376	-3.352551	-1.169684
B	2.239606	2.213026	1.332100
B	10.627079	-1.567652	1.611066
B	-0.173440	2.117675	2.856920
B	8.464874	-2.094782	3.414559
B	-3.248792	1.810684	0.499101
B	4.930673	-1.195136	1.760641
B	-3.031934	-1.057955	0.126637
B	4.815081	-4.095014	0.427978
B	-0.324184	-1.847364	-3.680146
B	6.793510	-3.604034	-3.745594
B	1.979236	-2.284243	-0.350425
B	9.380697	-5.580995	-1.426914
B	-1.863985	-1.283479	2.623657
B	6.229784	-5.023611	2.462439
B	0.438319	1.753677	-2.663658
B	8.163974	-0.356899	-1.864250
B	0.551544	-2.700963	-0.956204
B	7.875078	-5.557309	-1.884757
B	-2.134118	2.891724	0.891003
B	6.374635	-0.617289	2.262167
B	-3.229095	-0.897751	1.865678
B	4.839560	-4.286776	2.097961

Atom	x	y	z
B	-1.649080	-1.452508	-2.628338
B	5.649556	-3.661899	-2.494721
B	-3.727939	0.337558	0.934220
B	4.070240	-2.817918	1.620235
Mg	-1.781095	-2.947652	-0.637215
Mg	5.698208	-5.673700	-1.064034
Mg	-2.187066	1.114923	-2.674403
Mg	5.230456	-0.460895	-1.552184
Mg	1.546556	-1.075405	-2.299595
Mg	9.082731	-3.425998	-2.528198
Mg	-2.122792	1.070666	2.548619
Mg	6.342635	-2.812210	3.093186
Mg	2.020707	-0.691709	3.434579
Mg	10.377900	-5.045436	2.579761
Mg	1.638194	2.631846	-0.820411
Mg	9.717484	-0.214143	-0.086729

8.3 $(Ca_6@B_{40})_2$

Atom	x	y	z
B	-2.082844	-1.138123	-2.187220
B	7.920476	-2.090563	-2.806046
B	-3.900610	-1.625412	1.665018
B	4.530319	-2.252265	-0.012082
B	-3.107010	-0.558150	-0.916829
B	6.623308	-1.265321	-1.888886
B	3.402853	0.625099	1.217788
B	11.768359	-3.133546	2.298146
B	2.835849	-2.289647	0.180544
B	10.825266	-5.302233	0.431981
B	0.352017	-2.816259	-1.694466
B	9.295164	-4.658976	-2.157995
B	0.845944	2.038967	1.930027
B	9.840592	-0.929855	2.572395
B	1.057043	1.994831	-2.008065
B	11.484769	-0.526864	-0.811352
B	-0.578490	2.283255	-1.703543
B	10.072844	0.360461	-1.038509
B	-0.742298	2.373928	1.487262
B	8.709726	0.043541	1.806357
B	-3.010289	2.619153	-0.258301
B	7.518732	1.486863	-0.414134
B	-0.866446	-1.518924	2.602491
B	6.933237	-3.659792	1.667871
B	-0.024217	-0.251841	3.394139
B	7.808627	-2.888680	3.017992
B	2.320329	1.252960	2.235347
B	10.752119	-2.211265	3.177390
B	3.775325	-0.630676	0.202820
B	11.835166	-4.232885	1.145071
B	-1.161976	-0.317576	-3.231255
B	9.337906	-1.558688	-3.308340
B	-1.822053	3.360214	0.646503
B	8.417010	1.494440	0.993333
B	1.461005	-2.743348	0.953762
B	9.184921	-5.540346	0.569556
B	1.066348	0.919953	3.173723
B	9.176304	-2.229939	3.499216
B	-2.838261	-2.208182	-1.208802
B	6.563393	-2.888310	-2.352397
B	-3.741045	-1.819430	0.087305
B	5.305880	-2.279177	-1.563620
B	-2.683731	-1.029353	2.481184
B	5.604091	-2.624478	1.221297
B	2.315966	2.254818	-0.828369
B	12.290284	-0.906241	0.671460
B	-1.221496	-2.618480	-1.360124
B	7.864088	-3.890963	-2.205169
B	0.013771	-2.783828	0.013041
B	8.309252	-4.855115	-0.740287
B	-0.524145	1.239438	2.743039
B	8.061219	-1.254857	2.711022
B	-1.590555	-0.096625	3.162657
B	6.609293	-2.120772	2.317925
B	0.037747	-2.527977	1.671426
B	7.746579	-4.853028	0.850797
B	1.606493	-2.876088	-0.693255
B	9.935969	-5.458803	-0.909187
B	2.570680	1.238506	-2.017248
B	12.622095	-1.761013	-0.635195
B	-0.166597	1.126788	-2.862273
B	10.541023	-0.655304	-2.292910
B	-1.431805	1.800053	-0.127646
B	8.523211	0.043790	-0.038265
B	-3.551449	-0.305514	0.789898
B	5.390211	-0.862567	-0.676188
B	-0.344073	-1.580517	-2.505306
B	9.395016	-3.140861	-2.753986
B	2.335232	1.898671	0.733174
B	11.554406	-1.428056	1.994465
B	-3.073968	0.945661	-0.180120
B	6.804638	0.052487	-0.868425
B	3.069571	0.773527	-0.467984
B	12.270540	-2.648319	0.762465
B	0.424235	-0.392431	-3.378318
B	10.774049	-2.177007	-3.010540

Atom	x	y	z
B	-1.718027	3.319969	-1.036123
B	9.163861	1.661864	-0.497905
B	1.494335	0.809336	-3.145823
B	11.965331	-1.569217	-2.104908
Ca	-2.839007	1.537361	-2.600900
Ca	8.277271	0.730092	-2.763064
Ca	0.569286	3.904474	-0.030371
Ca	10.944313	1.142262	1.295262
Ca	-3.159615	1.755694	2.219102
Ca	6.094329	0.372388	1.540680
Ca	2.778315	-1.404199	-2.310856
Ca	11.877977	-4.103469	-1.582665
Ca	-2.226108	-3.455084	1.167438
Ca	5.645831	-4.566119	-0.541392
Ca	2.348364	-1.304396	2.664535
Ca	9.756411	-4.643890	2.759746

8.4 $(Sr_6@B_{40})_2$

Atom	x	y	z
B	0.957685	2.765093	-2.437945
B	7.864889	1.844161	-0.171623
B	1.055642	-1.576752	-3.337234
B	7.980509	-2.242725	-1.748540
B	-0.993797	0.420036	-2.868363
B	5.912755	-0.361161	-0.934001
B	0.269815	-2.384322	3.020578
B	7.477955	-4.205988	4.354057
B	1.684862	0.131435	2.971189
B	9.037883	-1.786300	4.666585
B	2.233095	1.347821	0.027396
B	9.445810	-0.010450	1.935100
B	-1.894109	-1.713546	1.308176
B	5.254189	-3.217258	2.908875
B	-1.372928	2.608798	2.270398
B	6.027161	0.988388	4.685968
B	-2.249627	2.431371	0.852937
B	5.098328	1.031710	3.264387
B	-2.628795	-2.278785	-0.068284
B	4.586640	-3.483466	1.437928
B	-2.221426	0.275312	-1.547631
B	4.774064	-0.590556	0.530810
B	3.029494	-1.694271	-0.889175
B	9.978271	-2.792695	0.602240
B	1.501917	-2.561376	-0.652023
B	8.536492	-3.671844	0.691483
B	-0.911514	-2.930592	2.072717
B	6.234675	-4.554020	3.420195
B	0.457278	-0.898035	3.582108
B	7.798062	-2.840717	5.149286
B	0.910575	4.019040	-1.524282
B	8.028090	2.878098	0.967215
B	-2.676553	-0.631759	0.176339
B	4.625070	-1.857684	2.031553
B	1.614508	-1.381655	2.336628
B	8.889496	-3.203936	3.815496
B	-0.005361	-3.173441	-0.553626
B	7.051282	-4.364694	0.626305
B	0.270548	1.474797	-3.151487
B	7.141515	0.738910	-1.105490
B	0.476038	-0.052931	-3.538349
B	7.351678	-0.727422	-1.725004
B	2.029438	-2.050138	-2.168007
B	9.060213	-2.862593	-0.730378
B	-1.039838	-0.007154	3.716299
B	6.325297	-1.926900	5.467817
B	1.364925	2.568436	-0.649292
B	8.448480	1.267250	1.533170
B	3.153947	-0.039893	0.155229
B	9.989275	-1.515191	1.601020
B	-1.338493	-3.246389	0.504869
B	5.794123	-4.590735	1.811860
B	0.389176	-2.373876	-1.973369
B	7.408968	-3.338418	-0.597192
B	2.329261	-1.482340	0.723766
B	9.353461	-2.979499	2.171988
B	1.836759	0.199153	1.263838
B	9.134454	-1.337211	3.039479
B	-0.907359	1.591752	3.519037
B	6.520690	-0.326496	5.591420
B	-0.837145	3.446868	0.963160
B	6.518956	2.031683	3.539053
B	-2.816980	-1.312066	-1.349930
B	4.102999	-2.283686	0.436557
B	-0.575428	-1.338274	-3.154773
B	6.356854	-2.079676	-1.437336
B	0.641887	1.114127	0.364130
B	7.915103	-0.218194	2.444077
B	-1.226356	-1.439731	2.836332
B	6.025054	-3.212643	4.422796
B	-2.097741	-0.913941	-2.717686
B	4.828669	-1.680222	-0.949489
B	0.454561	0.752022	3.884856
B	7.864544	-1.281921	5.733865
B	0.292282	3.944391	-0.100299
B	7.532889	2.610422	2.414057

Atom	x	y	z
B	-2.414928	1.052864	-0.058751
B	5.065011	-0.236793	2.178275
B	-0.844961	1.578861	0.699837
B	6.513123	0.182135	3.087586
Sr	-1.707366	3.004166	-1.844039
Sr	5.211511	2.040100	0.646723
Sr	-3.254643	0.506967	2.518084
Sr	3.936006	-1.099972	4.560148
Sr	-1.955368	-3.619676	-2.325045
Sr	4.935374	-4.408495	-1.056609
Sr	1.399779	2.830678	2.465085
Sr	8.804856	1.031534	4.697012
Sr	2.917044	0.572617	-2.586188
Sr	9.756514	-0.165782	-0.764150
Sr	1.703715	-4.062452	1.575109
Sr	8.499246	-5.662262	2.443108

8.5 $(Li_6@B_{40})_2$

Atom	x	y	z
B	2.744568	-1.035222	1.544708
B	10.312410	-0.189590	3.649221
B	1.699698	-0.971389	-2.659382
B	8.108432	-0.027601	0.092382
B	2.268469	-1.992198	0.181100
B	10.118771	-1.118745	2.238851
B	-2.969947	1.440543	-1.207294
B	4.016501	0.847130	2.805176
B	-1.394116	2.956123	1.242488
B	5.805386	2.396897	5.065885
B	1.853379	2.626919	1.229514
B	8.943052	2.765827	4.073923
B	-2.492247	-1.315104	-1.831546
B	5.504447	-1.997731	1.950671
B	-1.897268	-1.331266	2.108512
B	6.602635	-1.890133	5.280293
B	-0.639233	-2.438410	2.408359
B	8.067948	-2.700057	5.042189
B	-2.003540	-2.863808	-1.487592
B	6.438770	-3.065846	1.096437
B	-0.371221	-3.180186	0.843877
B	8.186803	-3.061274	3.423732
B	1.407544	1.710407	-1.669966
B	7.713724	2.537458	1.324724
B	0.131286	1.879097	-2.800872
B	6.103865	2.516755	0.735633
B	-2.200516	0.307491	-2.360227
B	3.941276	0.364580	1.080787
B	-2.208285	2.299249	-0.066604
B	4.963663	1.635577	3.855722
B	1.426768	-0.110494	2.329999
B	9.349314	0.013466	4.949268
B	-1.906701	-3.366647	0.034372
B	6.430597	-3.255603	2.739529
B	-0.626060	2.959941	-0.227011
B	6.081626	3.003257	3.562995
B	-0.776770	0.527430	-3.209609
B	5.375989	1.039802	0.364846
B	2.977726	-0.518907	-0.111934
B	9.606982	0.495844	2.331274
B	2.230277	0.271695	-1.617401
B	8.695311	1.224280	1.082969
B	0.816580	0.405656	-2.608109
B	7.030226	1.185677	0.436498
B	-3.041605	0.731191	0.275843
B	4.573884	-0.090398	4.038345
B	2.652876	1.286782	1.873989
B	9.760280	1.339372	3.885412
B	1.131787	2.826300	-0.240724
B	7.754550	3.230554	3.039490
B	-0.917464	-2.088152	-2.351484
B	6.033464	-1.745540	0.338958
B	0.073065	-1.027747	-3.129897
B	6.427760	-0.247761	-0.147065
B	0.263052	2.975995	-1.614576
B	6.476720	3.442651	2.022289
B	0.243495	2.844605	1.289631
B	7.390454	2.663120	4.625407
B	-2.749002	-0.002469	1.711198
B	5.376075	-0.829902	5.270588
B	-0.036703	-0.998609	2.804501
B	8.331389	-1.226119	5.663604
B	-0.420804	-3.053678	-0.870777
B	7.944873	-3.299031	1.812906
B	2.541327	-1.381284	-1.357165
B	9.465139	-0.326228	0.902612
B	1.047773	1.621558	2.296468
B	8.623199	1.615126	5.224176
B	-3.297424	-0.120548	-1.196690
B	4.290573	-0.892847	2.369267
B	0.964720	-2.990109	-0.055079
B	9.240937	-2.551667	2.238464
B	-2.098996	1.494504	1.534903
B	5.516612	0.795164	5.262961
B	0.134605	0.574967	3.147212
B	8.305383	0.328013	6.199588

Atom	x	y	z
B	-1.911130	-2.896438	1.564930
B	6.727919	-3.277961	4.352944
B	-1.377449	0.257015	2.543175
B	6.800262	-0.296155	5.867820
Li	1.516611	-2.808597	2.099208
Li	10.122140	-2.251619	4.301481
Li	-3.462778	-1.788212	0.262524
Li	4.704087	-2.502960	3.993359
Li	1.263653	-3.057319	-2.365064
Li	8.346141	-2.245308	-0.008900
Li	-0.851316	2.550837	3.301502
Li	6.950986	1.897389	6.830003
Li	3.373227	1.981422	-0.463081
Li	9.846922	2.844380	2.056216
Li	-1.788260	2.712780	-2.501518
Li	4.268868	3.002040	2.052661

8.6 $(Na_6@B_{40})_2$

Atom	x	y	z
B	0.498951	-3.586466	0.950517
B	0.937191	3.670297	-1.769763
B	-2.764425	-1.865386	-1.361955
B	-1.341491	5.886585	1.249866
B	-1.088858	-3.283407	1.155853
B	1.099233	4.102564	1.315563
B	1.256178	3.150608	-0.233645
B	-0.616158	9.915536	-1.759229
B	2.894187	0.847498	-1.254007
B	-1.256612	8.184629	-3.823745
B	2.340845	-1.936108	-1.087092
B	-0.805927	5.510408	-3.304254
B	-2.334679	1.646003	0.805982
B	0.823570	9.354198	0.692869
B	1.583932	1.108107	2.541087
B	2.578617	8.708055	-2.504070
B	0.403217	0.357702	3.458533
B	3.557479	7.965743	-1.349791
B	-3.454207	0.410957	0.870719
B	0.739520	8.525004	2.094045
B	-2.000085	-1.104006	2.741260
B	2.842587	6.742180	1.293248
B	-0.491693	-0.602479	-3.503432
B	-3.672721	6.843943	-0.792262
B	-1.848555	0.156138	-3.168904
B	-3.211848	7.927247	0.341065
B	-1.945136	2.212904	-0.863077
B	-0.815608	9.684835	-0.085872
B	1.992082	2.265889	-1.545636
B	-1.659903	9.574343	-3.021143
B	1.839125	-2.787048	1.425182
B	0.920498	5.017804	-2.802257
B	-2.279827	0.479279	2.091435
B	2.103722	8.311489	1.153447
B	1.934852	0.983950	-2.643893
B	-2.720013	8.337085	-3.005158
B	-2.463031	1.253175	-2.112495
B	-2.131118	9.099799	0.733412
B	-0.342089	-3.211030	-0.392047
B	-0.600997	3.358547	-0.916843
B	-1.413813	-2.679649	-1.474110
B	-0.668506	4.364842	0.948555
B	-1.588833	-1.401306	-2.537293
B	-2.761900	6.284061	0.437814
B	1.667082	2.536551	1.547817
B	1.177182	9.676692	-2.172914
B	1.370880	-2.898220	-0.241262
B	-0.497100	4.294870	-2.318291
B	2.119419	-0.569204	-2.013157
B	-2.174245	6.705197	-3.421579
B	-2.965147	0.814649	-0.625712
B	-0.729229	8.860129	1.483530
B	-2.887228	-0.330895	-1.908107
B	-1.833705	7.477368	1.152204
B	1.041867	-0.183795	-3.240467
B	-3.438809	7.043929	-2.397019
B	3.257214	-0.687692	-0.743307
B	-0.816898	6.725205	-4.415180
B	2.893797	1.470726	1.518865
B	1.490887	9.170118	-3.691545
B	1.718502	-0.455148	3.158168
B	3.099966	7.039513	-2.573493
B	-2.893460	-1.044964	1.281330
B	1.319143	6.981147	1.998261
B	-2.173912	-2.364033	0.159347
B	0.247859	5.593772	1.640221
B	2.677063	-1.492493	0.638882
B	0.691816	6.326342	-3.934935
B	-1.597818	3.098174	0.445460
B	0.634336	10.161474	-0.719635
B	-2.266039	-2.454146	1.828442
B	1.934302	5.476374	1.714458
B	2.363684	1.878749	0.023497
B	-0.105855	9.441098	-3.454276
B	2.510015	-1.649367	2.287031
B	2.165703	5.952173	-3.325852

Atom	x	y	z
B	-1.197724	0.139322	3.303797
B	3.418234	7.803164	0.232778
B	2.819606	-0.084488	1.927947
B	1.877305	7.554259	-3.631855
Na	0.061525	-2.289661	3.381275
Na	2.995871	5.308174	-0.771905
Na	-0.718730	2.559460	2.866358
Na	3.144258	10.242392	-0.376699
Na	-4.965405	-1.119930	-0.345254
Na	-0.781064	7.166716	3.545201
Na	4.993273	0.596722	0.500160
Na	0.522097	8.266082	-5.828933
Na	0.737750	-2.751331	-2.881987
Na	-2.959894	4.612214	-1.666632
Na	-0.239664	2.012226	-3.640158
Na	-3.776465	9.605863	-1.283351

8.7 $(K_6@B_{40})_2$

Atom	x	y	z
B	6.127719	3.631493	4.494496
B	4.678438	3.612605	11.020472
B	4.919763	1.774772	0.946066
B	3.066737	2.340726	7.134757
B	6.035529	2.102550	3.748023
B	4.564406	2.282988	10.041844
B	0.304430	3.908442	2.035154
B	-1.396205	4.560854	9.534144
B	1.601343	6.565400	3.399935
B	0.377267	6.633449	11.271961
B	4.893557	6.342776	3.020159
B	3.584474	6.696151	10.628463
B	0.953681	1.159859	2.457517
B	-1.134334	1.706201	9.329513
B	2.068211	3.046109	5.298373
B	0.878112	2.606618	12.912322
B	3.527961	2.073887	5.304664
B	2.329496	1.747763	13.211051
B	1.702963	-0.038879	3.337747
B	-0.092630	0.655323	9.942508
B	3.806267	0.159367	5.167784
B	2.237383	1.117771	11.499332
B	4.305708	4.601620	0.584176
B	2.842156	5.428192	7.332802
B	2.869507	4.082088	-0.183992
B	1.377708	4.783103	7.465091
B	0.970977	2.446065	1.309002
B	-0.669508	3.178850	8.491325
B	0.916926	5.251039	2.645306
B	-0.521392	5.690790	10.275389
B	5.099937	4.536798	5.350490
B	3.646385	4.233847	12.158163
B	2.193799	-0.074755	4.925446
B	0.624474	0.434916	11.342892
B	2.222364	6.078570	1.900972
B	0.900979	6.513595	9.659867
B	2.202393	2.568373	0.162989
B	0.123940	3.586932	6.975923
B	5.810227	3.548298	2.874189
B	3.746519	3.711327	9.628939
B	5.301443	3.435892	1.242126
B	2.778443	3.911035	8.278829
B	3.827601	2.887520	0.441913
B	1.164274	3.262011	8.254541
B	0.396139	3.898286	3.708314
B	-0.997672	4.008570	11.093089
B	5.620131	5.020206	3.731999
B	4.133241	5.118739	10.757206
B	3.934133	6.006966	1.705083
B	2.535620	6.825257	9.387476
B	2.578709	0.583664	2.067822
B	0.466220	1.668219	8.654746
B	3.314790	1.320809	0.743083
B	1.164944	2.005941	7.135286
B	2.919985	5.557040	0.498151
B	1.563480	6.362890	8.192458
B	3.266246	6.507031	3.214863
B	1.976743	6.838953	11.020145
B	0.606136	4.064519	5.351689
B	-0.289485	3.674507	12.524800
B	3.617289	3.633393	5.838939
B	2.631801	3.342029	13.208173
B	3.250348	0.541900	3.608390
B	1.596219	0.891284	9.969756
B	6.002982	2.069378	2.095009
B	3.772113	2.189024	8.669239
B	4.339770	6.013386	4.507783
B	3.142832	5.948228	12.009237
B	0.164589	2.500371	2.757470
B	-1.760579	3.084781	9.884721
B	4.685617	1.192980	4.287525
B	3.154774	1.279289	10.145608
B	1.058683	5.420137	4.423962
B	0.001450	5.174163	11.928174
B	3.844095	5.448946	5.970943
B	2.655432	4.994856	13.252243

Atom	x	y	z
B	2.069268	1.368382	5.906038
B	1.070582	0.899793	12.808363
B	2.188980	4.745507	5.793023
B	1.154553	4.340230	12.953482
K	6.105348	1.679991	6.789087
K	5.045137	1.267849	12.716268
K	-0.687400	1.038537	5.160991
K	-1.957405	1.220128	12.181223
K	5.306942	-0.771696	2.100251
K	2.345565	-0.829127	7.766040
K	2.430675	8.043929	5.777939
K	1.241388	7.387447	13.900856
K	7.011808	5.746533	1.131204
K	5.549237	5.903328	8.341187
K	0.222089	4.943017	-0.546135
K	-1.256066	6.134525	7.067750

8.8 $(Rb_6@B_{40})_2$

Atom	x	y	z
B	6.143904	3.641534	4.445089
B	4.659738	3.625948	11.079734
B	4.894900	1.768395	0.915177
B	3.122991	2.346243	7.154042
B	6.049639	2.111606	3.703784
B	4.569177	2.302791	10.091038
B	0.296252	3.897469	2.035619
B	-1.385425	4.564276	9.505010
B	1.585199	6.571508	3.384899
B	0.359362	6.623780	11.283216
B	4.873998	6.344103	2.981906
B	3.579241	6.717470	10.717693
B	0.948030	1.150423	2.459155
B	-1.121080	1.710013	9.288983
B	2.071577	3.026594	5.353063
B	0.838928	2.588245	12.908849
B	3.559790	2.071547	5.310843
B	2.291458	1.735893	13.229005
B	1.717547	-0.042052	3.332324
B	-0.084505	0.659091	9.909156
B	3.837756	0.165723	5.140753
B	2.223496	1.123910	11.507764
B	4.268974	4.594710	0.559536
B	2.884578	5.433596	7.328724
B	2.824180	4.074737	-0.195350
B	1.417024	4.808250	7.487627
B	0.953401	2.429622	1.307070
B	-0.638960	3.188790	8.472402
B	0.903392	5.244813	2.639929
B	-0.518984	5.690100	10.264213
B	5.097945	4.528521	5.298569
B	3.610053	4.233600	12.208893
B	2.223079	-0.082813	4.915545
B	0.614866	0.439908	11.321918
B	2.196952	6.076767	1.882339
B	0.920782	6.513655	9.677355
B	2.172314	2.552675	0.144231
B	0.171703	3.626470	6.978337
B	5.804725	3.550801	2.829455
B	3.759110	3.732902	9.666018
B	5.277048	3.435669	1.203982
B	2.814697	3.934727	8.301232
B	3.793889	2.887694	0.434855
B	1.197210	3.282070	8.265814
B	0.401335	3.895089	3.705932
B	-1.009033	3.995971	11.066280
B	5.613075	5.024857	3.688977
B	4.119815	5.133895	10.824346
B	3.907312	6.008273	1.672252
B	2.556374	6.848789	9.453479
B	2.575514	0.587187	2.053467
B	0.496161	1.696007	8.647637
B	3.291745	1.311285	0.710116
B	1.238022	2.035364	7.137979
B	2.888093	5.558046	0.470143
B	1.632935	6.376732	8.228811
B	3.247800	6.497487	3.189484
B	1.960191	6.846407	11.066485
B	0.633184	4.072425	5.341524
B	-0.323671	3.656718	12.507610
B	3.639601	3.627599	5.856738
B	2.587540	3.331094	13.241154
B	3.260475	0.553950	3.590364
B	1.602150	0.920133	9.968003
B	5.989369	2.069444	2.052384
B	3.804149	2.205509	8.702147
B	4.335316	6.008128	4.475424
B	3.114723	5.951154	12.076529
B	0.163788	2.490374	2.763352
B	-1.760976	3.087133	9.839584
B	4.701955	1.208498	4.259117
B	3.158476	1.303188	10.169149
B	1.065773	5.421274	4.413117
B	-0.022358	5.157449	11.920238
B	3.852992	5.447753	5.943691
B	2.603573	4.983112	13.297663

Atom	x	y	z
B	2.117746	1.343670	5.922471
B	1.044349	0.883046	12.800710
B	2.210135	4.745760	5.805104
B	1.111348	4.323645	12.964884
Rb	6.296664	1.576019	6.840115
Rb	5.142708	1.178537	12.865736
Rb	-0.838550	0.971226	5.255348
Rb	-2.136413	1.121306	12.214018
Rb	5.345317	-0.945911	2.034522
Rb	2.324127	-1.057475	7.805080
Rb	2.509790	8.266951	5.786135
Rb	1.149225	7.491283	14.062636
Rb	7.119165	5.816608	0.989248
Rb	5.725306	5.958324	8.368492
Rb	0.051224	4.961123	-0.676356
Rb	-1.320596	6.260232	6.942500

8.9 $(Sc_6@B_{40})_2$

Atom	x	y	z
B	-0.383123	3.864778	-0.040613
B	6.010236	4.131554	3.267139
B	0.691896	0.971070	3.052875
B	6.988928	1.126825	6.287486
B	-0.916387	3.481037	1.402741
B	5.423982	3.700861	4.698483
B	0.134383	-3.687603	-0.617982
B	6.656812	-3.655150	2.771104
B	2.167338	-1.620117	-1.855040
B	8.630493	-1.578731	1.548810
B	1.387569	2.439240	-1.952389
B	7.912837	2.861011	1.493332
B	-0.946998	-3.109752	1.969875
B	5.342002	-2.891198	5.240366
B	-1.771865	-0.679247	-2.504575
B	4.630385	-0.773196	0.556607
B	-2.299128	0.689754	-1.642295
B	4.264676	0.572212	1.692565
B	-2.072095	-1.901586	2.032993
B	4.202713	-1.738479	5.362139
B	-2.461091	0.914225	1.460496
B	3.955430	1.085374	4.852031
B	2.489730	0.833617	2.061589
B	8.674420	0.915517	5.288111
B	1.840810	-0.778838	1.709721
B	9.165343	-0.656331	5.438581
B	0.230787	-3.866952	1.038711
B	6.603727	-3.669355	4.406517
B	1.191084	-2.860164	-1.654506
B	7.707156	-2.884623	1.729784
B	-0.407342	3.056278	-1.481392
B	6.205167	3.033394	1.886618
B	-2.853249	-0.744898	1.133732
B	3.267161	-0.619411	4.545302
B	2.713341	-0.162768	-1.052302
B	9.012349	-0.122803	2.384401
B	1.772880	-1.963051	2.761648
B	8.080381	-1.689138	6.140931
B	0.790708	3.198572	1.005877
B	7.123874	3.384996	4.325631
B	-0.096476	2.309091	2.347874
B	6.217878	2.508274	5.659078
B	1.567760	2.261828	2.153136
B	7.842720	2.332851	5.361815
B	-1.601531	-3.467444	-1.101957
B	4.816869	-3.489373	2.157054
B	1.058953	3.417326	-0.664776
B	7.486847	3.836776	2.715651
B	2.533682	1.215378	-2.027259
B	8.403710	1.317008	1.284806
B	0.714613	-3.090938	2.361724
B	7.046831	-2.783540	5.652476
B	1.942127	-0.304429	3.385446
B	8.071884	-0.102912	6.560780
B	2.927776	-0.137250	0.623089
B	9.336426	0.036393	3.976998
B	2.233585	-0.227687	-2.675708
B	8.651049	-0.226019	0.711865
B	-1.647121	-2.294570	-2.274252
B	4.845626	-2.432351	0.907616
B	-1.527324	2.096897	-2.177312
B	5.139737	1.963315	1.175453
B	-1.972331	-0.353948	2.480058
B	4.204608	-0.180996	5.910386
B	-1.005227	0.940242	2.856992
B	5.242034	1.085022	6.046959
B	1.023328	0.909857	-2.844460
B	7.419222	0.572286	0.047439
B	-1.280115	-3.812394	0.411185
B	5.139208	-3.741929	3.702424
B	-1.769074	2.314736	2.097506
B	4.563139	2.552407	5.372982
B	-0.324090	-3.251606	-2.187765
B	6.211043	-3.251877	1.202081
B	-0.063463	2.123519	-2.900387
B	6.756581	1.924926	0.682282

Atom	x	y	z
B	-2.755272	0.320562	-0.099427
B	3.636568	0.448098	3.296689
B	-0.768433	0.600977	-2.724245
B	5.768591	0.551015	0.231677
Sc	-2.474778	2.614848	-0.164466
Sc	3.954095	2.753577	3.045788
Sc	-0.856320	-1.383695	-0.122215
Sc	5.292020	-1.367148	3.152283
Sc	-0.254690	-1.230791	3.532678
Sc	5.982659	-1.075949	6.899057
Sc	0.300932	-1.409169	-3.361994
Sc	6.840749	-1.563440	-0.140757
Sc	2.897597	2.216512	-0.010619
Sc	9.118757	2.263376	3.389463
Sc	2.401750	-2.576495	0.476423
Sc	8.647970	-2.338071	3.863285

8.10 $(Ti_6@B_{40})_2$

Atom	x	y	z
B	2.309550	-2.439062	1.123235
B	5.678571	4.540349	2.057375
B	0.051307	-0.229618	4.132963
B	3.436361	6.815796	4.984878
B	2.431171	-1.142014	2.043126
B	5.825619	5.818977	2.997571
B	-1.679504	1.797611	-1.371608
B	1.829763	8.963888	-0.476528
B	-2.613777	-0.623383	-2.569898
B	1.002732	6.379449	-1.612692
B	-0.084107	-2.993238	-0.403853
B	3.617325	3.747166	0.183773
B	0.745152	3.243578	-0.522063
B	4.278247	10.263512	0.305876
B	1.181959	0.042586	-3.487687
B	4.770218	7.112009	-2.586257
B	2.275931	-0.211706	-2.174621
B	5.872189	6.908450	-1.257961
B	0.646980	2.993247	1.200276
B	4.178010	9.864004	1.944071
B	2.939289	1.033753	0.579425
B	6.526889	8.211424	1.497504
B	-2.583802	-1.007879	2.127269
B	0.691692	6.245676	3.083907
B	-2.546100	0.333744	3.013780
B	0.880820	7.568014	3.951366
B	-0.855749	2.479710	-0.022897
B	2.718059	9.628473	0.852381
B	-2.743887	0.605101	-1.488716
B	0.841074	7.689064	-0.606716
B	1.677450	-2.553379	-0.423176
B	5.266761	4.362581	0.451189
B	2.347903	2.387254	-0.323059
B	5.514010	9.275073	0.709467
B	-2.824735	-0.983225	-0.901258
B	0.701684	6.182347	0.050687
B	-1.655450	1.697216	2.786375
B	1.827188	8.888832	3.704848
B	1.081239	-2.245703	2.284713
B	4.390323	4.786051	3.146143
B	0.056581	-1.716197	3.449973
B	3.350484	5.321513	4.300846
B	-1.393938	-0.913878	3.314130
B	1.965146	6.239087	4.184350
B	-0.746140	2.147655	-2.858258
B	2.783455	9.164390	-1.967101
B	0.807765	-3.160264	0.920853
B	4.104147	4.033618	1.683988
B	-1.787635	-2.395349	-0.460391
B	0.908291	4.610793	0.571087
B	-0.914136	2.414259	1.584633
B	2.602000	9.557174	2.481428
B	-1.249012	0.712046	4.008241
B	2.212622	7.866017	4.912519
B	-2.676795	-1.552249	0.628322
B	0.538344	5.778406	1.605410
B	-2.245455	-2.094919	-2.109450
B	1.314464	4.847782	-1.124648
B	-0.175285	0.884201	-3.653362
B	3.382881	7.896534	-2.735078
B	2.345915	-1.946234	-1.795646
B	5.919324	5.205239	-0.827211
B	2.155759	2.117087	1.585515
B	5.602413	9.089808	2.487566
B	1.290485	-0.424301	3.055611
B	4.629770	6.580430	3.867927
B	-0.894135	-2.889562	-1.851484
B	2.799808	3.846235	-1.256384
B	-0.506896	2.980876	-1.554142
B	3.043425	10.108908	-0.732291
B	2.231012	0.503230	2.040493
B	5.714788	7.462354	2.890376
B	-1.654668	0.678735	-2.862297
B	1.947471	7.660408	-1.937841
B	0.689899	-2.483439	-1.965074
B	4.361078	4.509501	-1.188941

Atom	x	y	z
B	2.662754	0.850680	-0.996206
B	6.289695	7.996817	-0.099820
B	1.466114	-1.487069	-3.078664
B	5.079272	5.606946	-2.148636
Ti	3.302509	-1.027401	-0.130082
Ti	6.711350	6.048456	0.938101
Ti	1.447163	2.106851	-2.410698
Ti	4.940681	9.071923	-1.504045
Ti	0.568487	1.764609	3.081416
Ti	4.034133	8.752417	3.923131
Ti	-0.660305	-1.301203	-3.486420
Ti	2.933493	5.653802	-2.573263
Ti	-1.076419	-2.637973	1.780116
Ti	2.155439	4.428681	2.594632
Ti	-2.390663	0.822459	0.758619
Ti	1.074707	8.074386	1.674939

8.11 $(B_{40})_2$ bonding along hexagonal ring

Atom	x	y	z
B	-0.475663	-2.814714	-1.383519
B	6.535979	-2.774085	-1.572302
B	3.060954	-0.193371	-2.191017
B	9.787248	-0.068200	-1.649491
B	1.133484	-2.354207	-1.159562
B	8.149454	-2.448300	-1.288508
B	-0.475634	2.814738	1.383523
B	6.535965	2.774088	1.572307
B	-2.987277	1.640858	-0.182234
B	3.739076	2.194939	-0.140489
B	-2.109023	-0.275811	-2.652690
B	4.773002	-0.100307	-2.407655
B	1.617480	1.111087	2.366049
B	8.603847	0.970743	2.286520
B	-1.350972	-1.161660	2.545625
B	5.735371	-1.301901	2.235317
B	-0.510049	-2.534951	2.037603
B	6.577668	-2.738243	1.954751
B	3.060951	0.193363	2.191006
B	9.787227	0.068201	1.649498
B	1.064625	-2.012497	1.625202
B	8.150983	-2.340515	1.533595
B	0.222339	1.517792	-3.002549
B	7.310076	1.630505	-2.814179
B	1.064638	2.012500	-1.625213
B	8.150985	2.340512	-1.533583
B	1.133506	2.354204	1.159545
B	8.149436	2.448294	1.288516
B	-1.803916	2.407119	0.613527
B	5.182529	2.506943	0.740179
B	-1.803939	-2.407087	-0.613514
B	5.182526	-2.506957	-0.740195
B	1.476211	-0.578578	2.583559
B	8.591718	-0.780381	2.375380
B	-1.791703	2.229906	-1.130861
B	5.323828	2.466178	-0.962922
B	2.027039	2.395027	-0.265262
B	8.908980	2.663898	-0.128823
B	0.263985	-1.974012	-2.504142
B	7.275630	-1.793614	-2.572921
B	1.617468	-1.111086	-2.366068
B	8.603883	-0.970753	-2.286524
B	1.476214	0.578578	-2.583574
B	8.591731	0.780378	-2.375367
B	-1.349464	1.644213	2.225058
B	5.666484	1.502533	2.151637
B	-1.349494	-1.644190	-2.225050
B	5.666487	-1.502551	-2.151644
B	-1.350966	1.161670	-2.545617
B	5.735388	1.301878	-2.235325
B	2.415333	1.051950	0.756784
B	9.479090	1.451043	0.803680
B	2.060799	0.738586	-1.036651
B	9.484798	1.379594	-0.954231
B	-0.510036	2.534962	-2.037606
B	6.577668	2.738232	-1.954751
B	-2.684831	0.734492	-1.508127
B	4.739344	0.912820	-0.886891
B	-2.108998	0.275837	2.652702
B	4.772999	0.100287	2.407649
B	-1.791720	-2.229886	1.130870
B	5.323843	-2.466190	0.962901
B	2.060820	-0.738620	1.036642
B	9.484803	-1.379604	0.954253
B	2.415337	-1.051988	-0.756811
B	9.479096	-1.451034	-0.803658
B	-2.679141	-1.013981	-1.312655
B	4.384649	-0.907319	-0.925385
B	0.264016	1.974033	2.504139
B	7.275610	1.793623	2.572936
B	2.027026	-2.395047	0.265245
B	8.908986	-2.663901	0.128837
B	-2.679118	1.014007	1.312669
B	4.384659	0.907304	0.925381
B	-2.987293	-1.640819	0.182254
B	3.739071	-2.194968	0.140475

Atom	x	y	z
B	0.222335	-1.517782	3.002542
B	7.310059	-1.630507	2.814181
B	-2.684839	-0.734462	1.508151
B	4.739338	-0.912837	0.886879

8.12 $(B_{40})_2$ bonding along heptagonal ring

Atom	x	y	z
B	-0.387253	-2.838211	-1.583957
B	-0.489147	-2.972420	5.323819
B	2.903971	-0.136105	-1.904616
B	-2.970095	-0.465581	5.351204
B	1.203314	-2.437147	-1.329271
B	-1.881430	-2.971313	6.156564
B	-0.010853	2.972421	1.376182
B	-0.112746	2.838211	8.283955
B	-2.613086	1.698159	-0.268309
B	2.263889	1.500459	6.604346
B	-1.973382	-0.227219	-2.790450
B	1.373935	-0.338449	4.014205
B	1.417078	1.740141	1.793784
B	-2.173794	1.070071	9.143500
B	-1.309595	-1.295748	2.534426
B	0.652490	-1.180358	9.438191
B	-0.407148	-2.722080	2.055101
B	-0.214283	-2.546656	8.967205
B	2.470093	0.465584	1.348793
B	-3.403973	0.136108	8.604614
B	1.195863	-2.173411	1.580714
B	-1.841973	-2.269288	8.759136
B	0.414574	1.450738	-3.191637
B	-0.912781	1.420656	3.759925
B	1.341970	2.269290	-2.059136
B	-1.695859	2.173409	5.119285
B	1.381428	2.971315	0.543437
B	-1.703314	2.437149	8.029268
B	-1.393847	2.436007	0.533196
B	1.144906	2.355007	7.417544
B	-1.644904	-2.355008	-0.717544
B	0.893847	-2.436008	6.166806
B	1.688331	-0.506797	2.309243
B	-2.226341	-0.645573	9.419867
B	-1.514538	2.313382	-1.265552
B	1.047844	2.137320	5.706204
B	2.230952	2.842209	-0.803859
B	-2.381817	2.507562	6.555916
B	0.377808	-2.010607	-2.709412
B	-0.855269	-2.549631	3.709688
B	1.673792	-1.070069	-2.443503
B	-1.917077	-1.740138	4.906215
B	1.726338	0.645575	-2.719868
B	-2.188328	0.506791	4.390760
B	-1.029446	1.829123	2.071687
B	0.705164	1.596287	9.072692
B	-1.205165	-1.596287	-2.372692
B	0.529449	-1.829126	4.628314
B	-1.152493	1.180359	-2.738189
B	0.809599	1.295745	4.165575
B	2.515105	1.764181	0.414351
B	-2.980881	1.363283	7.587306
B	2.685182	1.409748	-1.399364
B	-2.778400	1.080936	5.886266
B	-0.285720	2.546658	-2.267204
B	-0.092849	2.722078	4.644899
B	-2.396270	0.807437	-1.603615
B	1.947057	0.654683	5.245851
B	-1.873931	0.338447	2.685795
B	1.473380	0.227219	9.490452
B	-1.547841	-2.137322	0.993797
B	1.014535	-2.313382	7.965554
B	2.278400	-1.080937	0.813730
B	-3.185185	-1.409746	8.099362
B	2.480881	-1.363282	-0.887309
B	-3.015107	-1.764181	6.285650
B	-2.491190	-0.924916	-1.416025
B	1.764721	-1.097760	5.488786
B	0.355271	2.549630	2.990313
B	-0.877809	2.010609	9.409410
B	1.881818	-2.507561	0.144081
B	-2.730955	-2.842207	7.503858
B	-2.264719	1.097758	1.211216
B	1.991190	0.924915	8.116026
B	-2.763887	-1.500461	0.095655
B	2.113085	-1.698160	6.968312

Atom	x	y	z
B	0.412786	-1.420659	2.940076
B	-0.914578	-1.450736	9.891637
B	-2.447054	-0.654686	1.454150
B	1.896268	-0.807438	8.303618
